# Genome-Wide Survey and Expression Profile Analysis of the Mitogen-Activated Protein Kinase (MAPK) Gene Family in *Brassica rapa*

**DOI:** 10.1371/journal.pone.0132051

**Published:** 2015-07-14

**Authors:** Kun Lu, Wenjin Guo, Junxing Lu, Hao Yu, Cunmin Qu, Zhanglin Tang, Jiana Li, Yourong Chai, Ying Liang

**Affiliations:** 1 College of Agronomy and Biotechnology, Southwest University, Beibei, Chongqing 400715, PR China; 2 Chongqing Rapeseed Engineering Research Center, Southwest University, Beibei, Chongqing 400715, PR China; 3 Engineering Research Center of South Upland Agriculture, Ministry of Education, Southwest University, Beibei, Chongqing 400715, PR China; 4 College of Life Sciences, Chongqing Normal University, Chongqing 401331, PR China; University of Delhi South Campus, INDIA

## Abstract

Mitogen-activated protein kinase (MAPK) cascades are fundamental signal transduction modules in plants, controlling cell division, development, hormone signaling, and biotic and abiotic stress responses. Although MAPKs have been investigated in several plant species, a comprehensive analysis of the *MAPK* gene family has hitherto not been performed in *Brassica rapa*. In this study, we identified 32 MAPKs in the *B*. *rapa* genome by conducting BLASTP and syntenic block analyses, and screening for the essential signature motif (TDY or TEY) of plant MAPK proteins. Of the 32 *BraMAPK* genes retrieved from the *Brassica* Database, 13 exhibited exon splicing errors, excessive splicing of the 5' sequence, excessive retention of the 5' sequence, and sequencing errors of the 3' end. Phylogenetic trees of the 32 corrected MAPKs from *B*. *rapa* and of MAPKs from other plants generated by the neighbor-joining and maximum likelihood methods suggested that BraMAPKs could be divided into four groups (groups A, B, C, and D). Gene number expansion was observed for *BraMAPK* genes in groups A and D, which may have been caused by the tandem duplication and genome triplication of the ancestral genome of the *Brassica* progenitor. Except for five members of the *BraMAPK10* subfamily, the identified *BraMAPKs* were expressed in most of the tissues examined, including callus, root, stem, leaf, flower, and silique. Quantitative real-time PCR demonstrated that at least six and five *BraMAPKs* were induced or repressed by various abiotic stresses and hormone treatments, respectively, suggesting their potential roles in the abiotic stress response and various hormone signal transduction pathways in *B*. *rapa*. This study provides valuable insight into the putative physiological and biochemical functions of *MAPK* genes in *B*. *rapa*.

## Introduction

In nature, plants are often challenged with a myriad of biotic and abiotic stresses, including pathogen infection, drought, salt, cold, and oxidative stresses. These unfavorable conditions adversely affect plant growth and productivity and result in considerable loss of crop yield. Plants have evolved a series of highly elaborate signaling networks and regulatory mechanisms that impart protection at the molecular, cellular, organ, plant, and population levels [1]. Research efforts from around the world have provided insight into the stress response pathways of model plants and crops, and identified a number of canonical genes and intracellular signaling pathways involved in adaptation to heat, cold, drought, salt, osmotic stresses and pathogen infection. As the best studied signal transduction modules associated with responses to extracellular stimuli, mitogen-activated protein kinase (MAPK) cascades are ubiquitously found in eukaryotes ranging from yeast to plants and animals [2,3]. This cascade consists of three consecutively acting protein kinases, MAPK kinase kinases (MAPKKKs), MAPK kinases (MAPKKs), and MAPKs [4]. During exposure to stress, the stimulated plasma membrane activates serine/threonine kinase MAPKKKs [5], which in turn phosphorylate two serine/threonine residues in a conserved S/T-X3-5-S/T motif of the MAPKK activation loop. MAPKs are sequentially activated when both threonine and tyrosine residues at the conserved T-X-Y motif are phosphorylated by MAPKKs [6]. The activated MAPKs ultimately phosphorylate various downstream substrates and regulate the growth, development, programmed cell death, hormone signaling, and stress responses of the organism [7–9].

In plants, MAPK signaling pathways play important regulatory roles in several biological processes, such as biotic and abiotic stress responses and hormone and developmental signaling pathways [10]. Considerable progress has been made in deciphering the functions of MAPK genes [2]. In *Arabidopsis thaliana*, the bacterial flagellin-derived peptide flg22 triggers a complete MAPK cascade MEKK1-MKK4/5-MAPK3/6, which in turn increases the expression of *WRKY22*/*29* and confers resistance to both bacterial and fungal pathogens [3]. Recent genetic evidence indicated that YODA-MKK4/5-MAPK3/6 plays an important role in coordinating the epidermal cell fate specification of stomata through phosphorylation of SPEECHLESS (SPCH) [11,12]. The RAF-like MAPKKK CTR1 acts as an unconventional MAPKKK, blocking MKK9–MAPK3/6 activation and simultaneously regulating ethylene signaling and camalexin biosynthesis and leaf senescence [13,14]. Molecular and genetic research placed MAPK4 in a stress signaling pathway regulated by reactive oxygen species (ROS) and involving the MEKK1–MKK1/MKK2–MAPK4 cascade, which negatively regulates MEKK2, a positive regulator of the SUMM2-mediated immune response [15–17]. A complex composed of MAPK4, its nuclear substrate MAP KINASE SUBSTRATE 1 (MKS1), and WRKY33 regulated the expression of the antibacterial camalexin biosynthetic enzyme PHYTOALEXIN DEFICIENT3 (PAD3), which indicates that MAPK4 is a negative regulator of defense responses [18–20]. The MEKK1-MKK2-MAPK4/6 cascade was shown to be activated by salt, drought, and cold stresses [21]. Furthermore, MAPK4 facilitates male-specific meiotic cytokinesis in *Arabidopsis* via the NACK2/TES/STUD-ANP2/3-MKK6-MPK4/11/13 signaling module [22–25]. A cascade that involves MKK1–MAPK6 participates in ABA and sugar signaling during seed germination and H_2_O_2_ accumulation through the ABA-induced expression of *CAT1*, *NCED3*, and *ABA2* [26]. *MAPK* genes in several important crops have also attracted considerable attention. *GhMPK7*, a group C MAPK in cotton (*Gossypium hirsutum*), is induced by pathogen infection and multiple defense-related signal molecules, and may be an important regulator of pathogen resistance and plant growth and development [27]. Overexpression of *Brassica napus MAPK4* and constitutive expression of an oxalate oxidase from *Triticum aestivum* enhances resistance to *Sclerotinia sclerotiorum* in transgenic *Brassica napus* [28]. Rice *MAPK* genes, *OsBIMK1 (Oryza sativa* L. *BTH-induced MAPK 1)* and *OsBIMK2*, play important roles in disease resistance against tomato mosaic virus and a fungal pathogen [29,30]. *OsMAPK33* and *OsMAPK44* were shown to be involved in salt tolerance through unfavorable ion homeostasis as negative and positive regulators, respectively [31,32]. Three maize (*Zea mays*) *MAPK* genes, *ZmMPK3*, *ZmMPK5*, and *ZmPMK17*, are involved in signal transduction pathways associated with different environmental stresses [33–35].

The increasing availability of plant genome sequences provides unprecedented opportunities to identify and examine the phylogenetic relationships of important gene families at the whole-genome level. Prompted by the biological and physiological significance of *MAPK* genes, and their potential applications for improving plant stress tolerance, the *MAPK* gene families have been identified and characterized in a variety of plant species. In the model plants *Arabidopsis* [3], rice [36,37], and purple false brome (*Brachypodium distachyon)* [38], a total of 20, 15, and 16 *MAPK* members were identified at the genome scale, while 10, 12, 15, 16, 17, and 17 homologs were found in mulberry (*Moraceae morus*) [39], grapevine (*Vitis vinifera*) [40], wheat (*Triticum aestivum*)[41], tomato (*Solanum lycopersicum*) [42], maize [43], and tobacco (*Nicotiana tabacum*) [44], and as many as 21, 25, and 26 putative *MAPK* genes were identified in poplar (*Populus trichocarpa*) [45], banana (*Musa acuminata*) [46], and apple (*Malus domestica*) [47], respectively.

*Brassica rapa* (*2n* = 20, AA) is a Brassicaceae species closely related to *Arabidopsis*, and is grown throughout the world for the production of condiments, vegetables, vegetable oils, and fodder [48]. In addition, as one of the diploid progenitor species of the major oilseed allotetraploid crops, *Brassica juncea* (*2n* = 36, AABB) and *Brassica napus* (*2n* = 38, AACC), it is a unique species for investigating the divergence of gene function, and genome evolution associated with polyploidy, extensive duplication, and hybridization [48,49]. During the growing season, various types of biotic (clubroot, white rust, and diamondback moth) and abiotic (heat, cold, and drought) stresses pose a major threat to *B*. *rapa*. The recently completed genome sequencing project for *B*. *rapa* provides an excellent opportunity to identify gene families and other functional elements involved in stress resistance, such as *thaumatin-like protein* (*BrTLP*) and *dirigent* (*BrDIR*) gene families [50–52]. To date, systematic investigations and functional analyses of *MAPK* gene families have not been reported for *Brassica* species, despite the importance of MAPK proteins in multiple biological processes.

In this study, we sought to provide a comprehensive overview of the *MAPK* gene family in *B*. *rapa*. To ensure the integrity of the gene structures and sequences, all the *B*. *rapa MAPK* (*BraMAPK*) genes with potential incorrect annotations in the *Brassica* Database (BRAD, http://brassicadb.org/brad/) were cloned based on the gene structure of *AtMAPK* genes [53]. Then, the chromosome location, gene structure, and protein motifs of the putative *BraMAPK* genes were carefully analyzed based on the corrected sequences. Additionally, the putative *BraMAPK* genes were subjected to phylogenetic analyses with counterparts from *Arabidopsis* and other known species to identify clusters of orthologous groups, enabling functional annotation. We also conducted a comprehensive analysis of all the identified *BraMAPK* genes to survey their tissue specificity and to determine which of these genes contribute to stress and hormone responses using quantitative real-time polymerase chain reaction (qRT-PCR) analysis. These data provide valuable insight into the classification and putative functions of the *BraMAPK* gene family. Ultimately, these findings may lay the foundation for genetically engineering *B*. *rapa* lines with improved stress tolerance.

## Materials and Methods

### Plant growth and treatments

Seeds of the *B*. *rapa* cultivar 'Chiifu-401-42' were kindly provided by the Chongqing Rapeseed Technology Research Center, China. Plant materials used to determine the stress- and hormone-induced expression pattern of *BraMAPK* genes were cultivated in an illuminated incubator with a thermo-photoperiod of 25°C for 16 h/18°C for 8 h (light/dark), 400 μM m^−2^ s^−1^ light intensity, and 60% relative humidity, as previously described [54].

To investigate the induced expression patterns of *BraMAPK* genes in the seedling leaves in response to various treatments, 4-week-old seedlings grown in soil were used. For osmotic or salt stress treatments, the roots of seedlings were soaked in solutions, without or with 10% (w/v) PEG-8000 (polyethylene glycol) or 200 mM NaCl for 10 min. For waterlogging stress treatment, the pots were placed in a container with deionised water maintained at the level of 2 cm above the soil surface. For cold and heat treatments, seedlings were maintained at 4°C and 37°C, respectively, in a light incubator. For hormone treatments, solutions containing 10 μM indole acetic acid (IAA), 10 μM *α*-naphthaleneacetic acid (NAA), 2.5 μM brassinolide (BR), 25 μM gibberellic acid (GA_3_), or 100 μM abscisic acid (ABA) were prepared in ethanol, and that containing 10 μM 6-benzyladenine (6-BA) was dissolved in 0.1 M NaOH using 0.1% triton X-100 and sprayed the leaves of 4-week-old seedlings. Plants treated with 0.1% triton X-100 in ethanol or NaOH were taken as controls. For wound stress treatment, the second youngest leaves were wounded with sterile pins, causing approximately five punctures with a diameter of 0.1 mm per cm^2^. The seedling leaves from both treated and control plants were harvested at 0 h, 0.5 h, 6 h, and 12 h after treatment. Three biological replicates were collected per time point, each comprising the second youngest leaves of three independent plants. Samples were immediately frozen in liquid nitrogen, and stored at −80°C.

### Nucleic acid isolation and cDNA synthesis

Total RNA was extracted from 100 mg of samples using the RNAprep Pure Plant Kit (Tiangen, Beijing, China), according to the manufacturer’s instructions. DNase digestion was performed with RNase-free DNase I (TaKaRa) to eliminate trace genomic DNA. The concentration and quality of RNA were assessed by agarose gel electrophoresis and a NanoDrop ND-2000 Spectrophotometer (Thermo Scientific, Waltham, MA, USA). The first-strand cDNA was synthesized using PrimeScript RT Master Mix (Perfect Real Time) (TaKaRa, Dalian, China) in a 20-μL reaction system containing 1 μg total RNA. Genomic DNA was isolated from young leaves using a cetyltrimethylammonium bromide (CTAB) method as previously described [54].

### Identification and correction of the *BraMAPK* members

Genomic and protein sequences of *B*. *rapa* were downloaded from BRAD [53]. Sequences of 20 *Arabidopsis* MAPK (AtMAPK) proteins were retrieved from The *Arabidopsis* Information Resource (http://www.arabidopsis.org/) [55] and used to search against *B*. *rapa* protein models with HMMER3 at a confidence cutoff e-value of < 1e-20. Then, BLASTP analysis with a threshold e-value of 1e-20 was carried out using AtMAPK proteins as query against 372 unique hits of HMMER3 [56,57]. To identify *BraMAPK* genes accurately from genome sequences, the unique sequences obtained from the abovementioned programs were further filtered based on the typical structural features of plant MAPK proteins as previously reported [58], ensuring that the 11 conserved subdomains in the kinase catalytic domain of the MAPK and the essential TDY or TEY signature motif in the activation loop were present.

The coding sequences (CDSs) of *BraMAPK* genes were retrieved from the BRAD based on the list of BraMAPK proteins, and the corresponding genomic sequences were extracted from the *B*. *rapa* genome sequence based on chromosomal location. Both CDS and genomic sequences of each *AtMAPK* gene and *B*. *rapa* ortholog were aligned using ClustalW in Geneious Pro 4.85 [59,60]. If significant differences in gene length, exon number, or exon/intron boundary were found between a *BraMAPK* gene and its *Arabidopsis* ortholog, those *BraMAPK* genes were regarded as sequence errors.

To amplify the CDSs of *BraMAPK* genes with sequence errors, standard 50-μl PCR reactions were carried out under standard conditions with 1 μl of first-strand cDNA. Thermal cycling was performed on a Veriti PCR thermal cycler (ABI) as follows: 94°C for 4 min, 35 cycles of 94°C for 1 min, annealing for 1 min and 72°C for 3 min, and succeeded by 72°C for 10 min. The corresponding genomic sequences were amplified using the template with 0.5 μg of total genomic DNA under the aforementioned PCR conditions. Gel-purified PCR products were cloned into pGEM-T vector (Promega). At least 3 PCR-positive colonies of each PCR fragment were sequenced on both strands to confirm the sequence. All primers used for gene sequence correction were designed according to the ClustalW2 alignment results and *B*. *rapa* genome sequence [51]. Primer sequences, product lengths and annealing temperatures for each primer pairs were listed in S1 Table.

### Phylogenetic analysis of AtMAPK and BraMAPK proteins

The sequences of MAPK proteins from *Arabidopsis*, *B*. *rapa*, rice, and maize, as well as several other well-known plant MAPK proteins were retrieved from previous reports and used for phylogenetic tree construction. Multiple alignments of the nucleotide and amino acid sequences were performed using ClustalW2. To construct an accurate phylogenetic tree, ProtTest v3.4 was used to select the best-fitting amino acid substitution model with the Akaike information criterion (AIC) [61]. The phylogenetic tree for the MAPK proteins was constructed using the neighbor-joining (NJ) method in the MEGA 6.0 program with the Jones, Taylor, and Thorton (JTT) model [62]. Gaps were treated as deletions in pairwise comparisons of sequences. The reliability of the tree was measured by bootstrap analysis with 1,000 replicates. Subsequently, a maximum likelihood (ML) tree was constructed with PhyML version 3.0.1 using the JTT+I+G model of amino acid substitution, an estimated gamma distribution parameter, and a bootstrap of 100 replicates [63]. All of the phylogenetic trees were visualized using the program FigTree v1.4.0 (http://tree.bio.ed.ac.uk/software/figtree/).

### Sequence analyses

The chromosomal distribution of all *BraMAPK* genes was determined based on the results of BLASTN analysis against the ten *B*. *rapa* chromosomes. MapInspect software (http://www.plantbreeding.wur.nl/uk/software_mapinspect.html) was subsequently used to draw the location images of *BraMAPK* genes. Exon-intron structure analysis of the *BraMAPK* genes was conducted and displayed by comparing CDSs and their corresponding genomic sequences using the Gene Structure Display Serve 2.0 (http://gsds.cbi.pku.edu.cn) [64]. The MEME program was used to statistically identify conserved motifs in the complete amino acid sequences of plant MAPK proteins [65]. For MEME, the default settings were used, except that the minimal and maximal width of the motif were set to 6 and 300 amino acids, respectively, and the maximum number of motifs to find was 100. Each motif was individually checked so that only motifs with an e-value of < 1e–10 were kept for motif detection in *Arabidopsis* and *B*. *rapa* MAPK proteins. The syntenic relationship between *AtMAPK* and *BraMAPK* genes was established using BRAD [53].

### Expression analysis of *BraMAPK* genes

To investigate the spatial expression patterns of *BraMAPK* genes, the RNA-seq data were downloaded from the NCBI Gene Expression Omnibus (http://www.ncbi.nlm.nih.gov/geo) under accession number GSE43245. The gene expression levels were quantified as FPKM (fragments per kilobase of exon per million fragments mapped) values by the TopHat/Cufflinks pipeline in the previous report [66,67]. The log_2_-transformed (FPKM + 1) values of the 32 *BraMAPK* genes were used for heat map generation.

To determine the expression profiles of *BraMAPK* genes in the second youngest leaves sampled from treated and control plants, quantitative real-time PCR (qRT-PCR) was performed using SYBR Premix Ex *Taq* II (Perfect Real Time) (TaKaRa, Dalian, China) in a CFX96 real-time PCR system (Bio-Rad, USA) with the following conditions: 95°C for 2 min and then 40 cycles of 95°C for 10 s, annealing for 30 s with designated temperature listed in S2 Table, and 72°C for 20 s. Gene-specific primers were designed using Geneious Pro 4.8.5 software with previously described parameters [59,68]. Using gradient PCR with the cDNA as template, the highest feasible annealing temperatures of primer pairs were determined and listed in S2 Table. To ensure primer specificity a BLASTN search against the whole *B*. *rapa* genome was performed, and agarose gel electrophoresis and melting curve analysis ensured that only one product was amplified. Three technical replicate reactions were implemented at each time point. All the qRT-PCR assays were repeated three times. Two endogenous housekeeping genes, *B*. *rapa UBC21* (*ubiquitin conjugating enzyme 21*) and *GAPDH* (*glyceraldehyde-3-phosphate dehydrogenase*), were used for normalization and quantification of fold changes of *BraMAPK* genes using the 2^–ΔΔCT^ method [69–71]. Genes with (1) ΔΔCt values of > 1 or < -1 and (2) *p*-value < 0.05 (determined by two-tailed Student's *t*-test) were considered as being significantly regulated. The sub-functionalization of duplicated members in the same *BraMAPK* gene subfamily were statistically measured by two-way analysis of variance (ANOVA).

To verify the consistency of expression levels of *BraMAPK* genes in leaves between qRT-PCR and RNA-seq methods, and compare the expression patterns of *MAPK* genes between *B*. *rapa* and *Arabidopsis*, the expression data of *AtMAPK* genes were obtained from AtGenExpress (http://www.weigelworld.org/resources/microarray/AtGenExpress) databases [72]. The relative expression levels of *BraMAPK* genes in leaves after 0 h of mock treatment in qRT-PCR analysis were calculated as Ct(IC)-Ct(X) ('Ct' stands for cycle number; 'IC' stands for two internal control genes; 'X' stands for *BraMAPK* genes), while those in RNA-seq analysis were calculated as Log_2_ (FPMK + 1) transformed expression value ((IC)-(X)). In *Arabidopsis*, the relative expression levels of *AtMAPK* genes in leaves were calculated as Log2 transformed expression value ((IC)-(X)) ('X' stands for *AtMAPK* genes). All the Pearson correlation coefficients were estimated by using SPSS 21 (IBM SPSS Statistics, IBM Corporation).

## Results

### Identification and correction of the 32 *BraMAPK* members in *B*. *rapa*

To obtain accurate sequences of *BraMAPK* genes from the *B*. *rapa* genome, we divided the whole analysis into two steps, identification and correction. Based on the multiple sequence alignment of 20 AtMAPK proteins, a new profile HMM file AtMAPK.hmm was built using the hmmbuild program in the HMMER3 package [56]. Then, 372 candidate proteins were identified by querying all *B*. *rapa* protein models against AtMAPK.hmmm using the hmmsearch program with an e-value cutoff of 1e-20. The amino acid sequences of candidate proteins in *B*. *rapa* were subjected to BLASTP against the *Arabidopsis* proteome, and 32 BraMAPK proteins were identified with top hits to AtMAPK orthologs (Table 1). Recent comparative genomics studies in Brassicaceae have recognized the existence of 24 conserved genomic blocks [73]. Syntenic orthologs between *Arabidopsis* and *B*. *rapa* were also extensively surveyed and determined by both sequence similarity and homozygosity of their flanking genes, and are listed in BRAD according to the gene order of *Arabidopsis* [53]. Of the 34 candidate BraMAPK proteins obtained with homology to 18 AtMAPK proteins (i.e., all AtMAPK proteins except AtMAPK4 and AtMAPK14), only 30 BraMAPK proteins were kept. Bra032622 and Bra022248 were discarded as their orthologs were AT1G01140 (CBL-INTERACTING PROTEIN KINASE 9) and AT3G28770 (Protein of unknown function 1216), and Bra016567 and Bra000411 were also abandoned since they encode very short polypeptides of 135 and 157 amino acids, respectively. A comparison of 30 candidate BraMAPK proteins retrieved from BRAD and BLASTP analyses showed that two additional BraMAPK proteins occurred outside the 24 syntenic blocks between *Arabidopsis* and *B*. *rapa*. Finally, a total of 32 BraMAPK proteins corresponding to 18 AtMAPK proteins were identified and used for further investigation. The BraMAPK proteins were named according to the nomenclature used for AtMAPK proteins (Table 1).

**Table 1:** Main structural features of the *BraMAPK* family members.

Nomenclature	Name in BRAD	Type of error	Length of original CDS (bp)	Length of corrected CDS (bp)	Exon number	Chr.	Subgenome	Triplication block	MAPK group	T-loop	Protein length (aa)
BraMAPK1	Bra019955		1110		2	A06	LF	A	C	TEY	369
BraMAPK2	Bra035437		1113		2	A01	MF1	D	C	TEY	370
BraMAPK3	Bra038281	A	1002	1113	6	A06	LF	M	A	TEY	370
BraMAPK4	Bra000955		1122		6	A03			B	TEY	373
BraMAPK5	Bra035233	D	1407	1122	5	A09	LF	P	B	TEY	373
BraMAPK6-1	Bra004784		1188		6	A03	LF	J	A	TEY	392
BraMAPK6-2	Bra000326		1179		6	A05	MF2	J	A	TEY	395
BraMAPK7-1	Bra037234		1107		2	A09	MF1	H	C	TEY	368
BraMAPK7-2	Bra039629		1107		2	A07	LF	H	C	TEY	368
BraMAPK8-1	Bra025929		1746		11	A06	LF	A	D	TDY	581
BraMAPK8-2	Bra031017	A	1587	1728	11	A09	MF2	A	D	TDY	575
BraMAPK9	Bra022276	C	1785	1506	11	A05			D	TDY	501
BraMAPK10-1	Bra003390	D	1190	1107	6	A07	MF2	N	A	TEY	368
BraMAPK10-2	Bra007476		1203		6	A09	LF	N	A	TEY	400
BraMAPK10-3	Bra007475		1170		6	A09	LF	N	A	TQY	389
BraMAPK10-4	Bra014528		1173		6	A04	MF1	N	A	TEY	390
BraMAPK10-5	Bra014527		1182		6	A04	MF1	N	A	TEY	393
BraMAPK12-1	Bra004959	A	1083	1119	6	A05	LF	J	B	TEY	372
BraMAPK12-2	Bra039292		1119		6	A04	MF1	J	B	TEY	372
BraMAPK13	Bra031597		1116		6	A09	MF2	A	B	TEY	371
BraMAPK15	Bra003834	B	1521	1734	2	A07	MF2	E	D	TDY	577
BraMAPK16-1	Bra002201	A	1512	1686	10	A10	LF	R	D	TDY	561
BraMAPK16-2	Bra006490		1677		10	A03	MF1	R	D	TDY	558
BraMAPK17-1	Bra026665	D	1785	1467	8	A02	MF1	K	D	TDY	488
BraMAPK17-2	Bra024886		1467		9	A06	LF	K	D	TDY	487
BraMAPK17-3	Bra017450		1464		8	A09	MF2	K	D	TDY	486
BraMAPK18-1	Bra038128	B	1698	1734	2	A05	MF2	C	D	TDY	577
BraMAPK18-2	Bra039676	B	1767	1803	2	A06	LF	C	D	TDY	600
BraMAPK19-1	Bra027317		1812		8	A01	LF	F	D	TDY	592
BraMAPK19-2	Bra021573		1779		8	A05	MF1	F	D	TDY	603
BraMAPK20-1	Bra000277	AD	1938	1815	8	A03	LF	J	D	TDY	604
BraMAPK20-2	Bra004727	A	1593	1809	10	A05	MF2	J	D	TDY	614

Type of error A: exon splicing error; B: excessive splicing of the 5' sequence; C: excessive retention of the 5' sequence; D sequencing error of the 3' end. Subgenome and triplication block information of *BraMAPK* genes were derived from BRAD.

Since divergence from the *Arabidopsis* lineage, the *Brassica* species have triplicated counterparts to the corresponding homologous segments in *Arabidopsis* [51]. The *B*. *rapa* genome has been classified into less fractionized (LF), more fractionized 1 (MF1), and more fractionized 2 (MF2) subgenomes according to the comparative analysis with the *Arabidopsis* genome and the gene orders and gene densities of the subgenomes [53]. Of the 30 *BraMAPK* genes in the 11 different triplicated blocks, 13, 8, and 9 occurred in the LF, MF1, and MF2 subgenomes of *B*. *rapa*, respectively. Six *AtMAPK* genes only possess one homologous copy in the syntenic blocks of *B*. *rapa*, while 1, 1, and 8 *AtMAPK* genes have 5, 3, and 2 homologous genes in the *B*. *rapa* subgenomes, respectively (Table 1).

The accuracy and reliability of gene family evolutionary characterization and expression profiling analysis depend on the availability of correct genomic sequences. To evaluate whether the original *BraMAPK* genes were accurate, we aligned the CDSs and genomic sequences of each *AtMAPK* gene with the corresponding homologous genes in *B*. *rapa* obtained from BRAD. Multiple alignments were carried out by ClustalW in Geneious Pro 4.85 [59]. The alignment results revealed that the gene length, exon number, and exon-intron boundary of 19 *BraMAPK* genes were the same as for *MAPK* orthologs in *Arabidopsis*, implying that these genes annotated by the *B*. *rapa* Genome Sequencing Consortium are correct and suitable for further analysis. However, the remaining 13 original *BraMAPK* genes from BRAD were found to be incorrect based on four different reasons (Table 1). Some exons in *Bra038281* (*BraMAPK3*), *Bra031017* (*BraMAPK8-2*), *Bra004959* (*BraMAPK12-1*), *Bra002201* (*BraMAPK16-1*), and *Bra004727* (*BraMAPK20-2*) were spliced as introns, while the sequences of the 5′ end of *Bra003834* (*BraMAPK15*), *Bra038128* (*BraMAPK18-1*), and *Bra039676* (*BraMAPK18-2*) were also spliced excessively, causing their original CDSs to be significantly shorter than the corresponding corrected sequences. Sequencing errors in the genomes often lead to gene finding and annotation problems. Sequence mutations in the 3′ ends of *Bra035233* (*BraMAPK5*), *Bra003390* (*BraMAPK10-1*), and *Bra026665* (*BraMAPK17-1*) caused incorrect longer CDSs, as did excessive retention of the 5′ sequence in *Bra022276* (*BraMAPK9*). Furthermore, two kinds of annotation errors were found in *Bra000277* (*BraMAPK20-1*), including a splicing error and a sequencing error in the 3′ end. After correction by manual cloning and sequencing, the sequences of 13 corrected *BraMAPK* genes supported at least two independent clones with the same sequencing results were deposited in the S1 File, and used for further phylogenetic, conserved sequence, and expression and functional divergence analyses.

### Phylogenetic relationship between BraMAPK and other plant MAPK proteins

**Fig 1:**
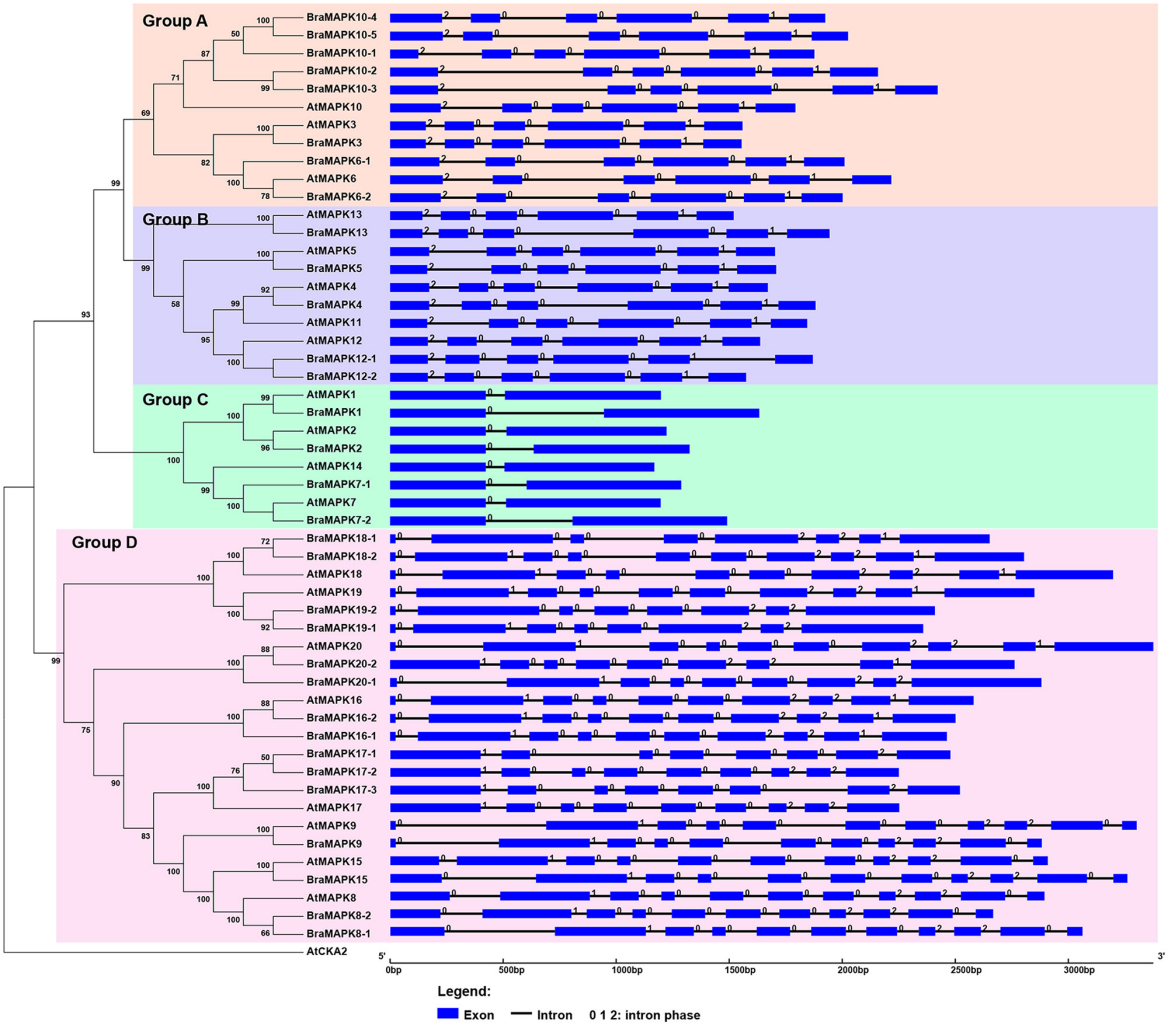
Phylogenetic analysis and gene structure of *MAPK* genes in *Arabidopsis* and *B*. *rapa*. Full-length protein sequences of 20 AtMAPKs, 32 BrMAPKs and AtCKA2 were aligned by using the ClustalW2 program. The phylogenetic tree (left panel) was constructed by using the MEGA 6.0 program and the neighbor-joining method (1000 bootstrap replicates), and displayed using FigTree v1.4.0. Only bootstrap values greater than 50% are denoted at the nodes. The 52 MAPK proteins in *A*. *thaliana* and *B*. *rapa* were clustered into four distinct groups (Groups A, B, C, and D). Gene structure is shown in the right panel. Exons and introns are shown by blue boxes and black horizontal lines, respectively. Introns in phases 0, 1, and 2 are represented by the numbers 0, 1, and 2, respectively. The scale bar represents 1.0 kb. At: *A*. *thaliana*; Bra: B. *rapa*.

**Fig 2:**
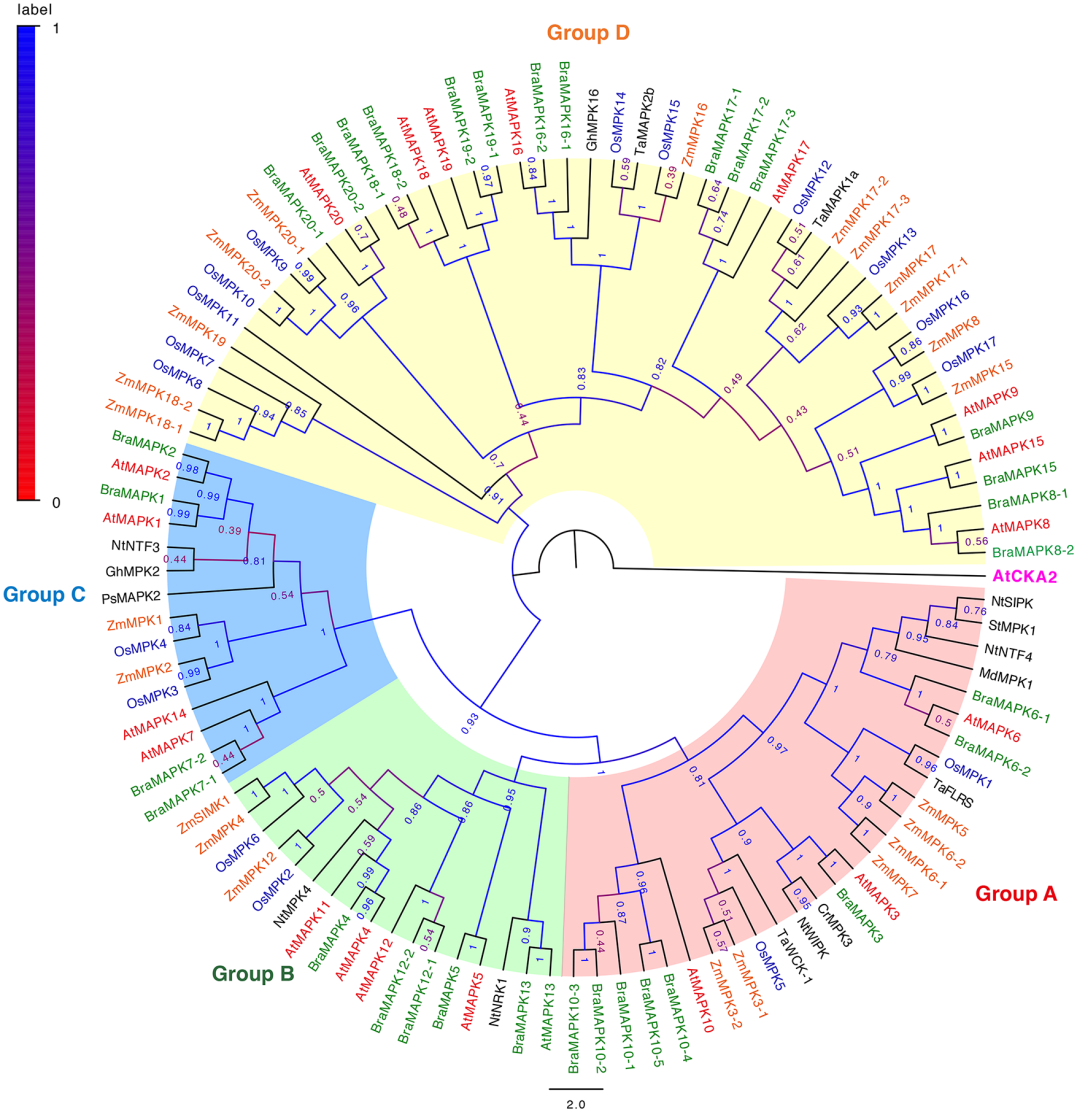
Phylogenetic relationships of plant *MAPK* proteins. The phylogenetic tree was generated by the NJ method with bootstrap analysis (1000 replicates) from the amino acid sequence alignment of MAPK protiens in *Arabidopsis*, *B*. *rapa*, rice, *Zea mays*, and other plants using MEGA 6.0 program. The tree was displayed with FigTree v1.4.0. Bootstrap values of >50% are denoted at the nodes. Plant MAPK proteins in the phylogenetic tree were clustered into four distinct groups (Groups A, B, C, and D). At: *A*. *thaliana*; Bra: *B*. *rapa*; Cr: *Catharanthus roseus*; Gh: *G*. *hirsutum*; Md: *M*. *domestica*; Nt: *N*. *tabacum*; Os: *O*. *sativa*; Ps: *Pisum sativum*; St: *Solanum tuberosum*; Ta: *T*. *aestivum*; Zm: *Z*. *mays*.

An accurate phylogenetic tree describes the estimated evolutionary relationships among various organisms or genes that take into account the time and divergence process from a common ancestor. In our study, ProtTest v3.4 was used to select the best-fitting candidate model, and the results of AIC suggested that JTT+I+G (-lnL = 35470.13) was the appropriate amino acid substitution model. As a result, two phylogenetic trees were constructed with the NJ and ML methods using two sets of MAPK protein sequences (Figs 1 and 2).

**Fig 3:**
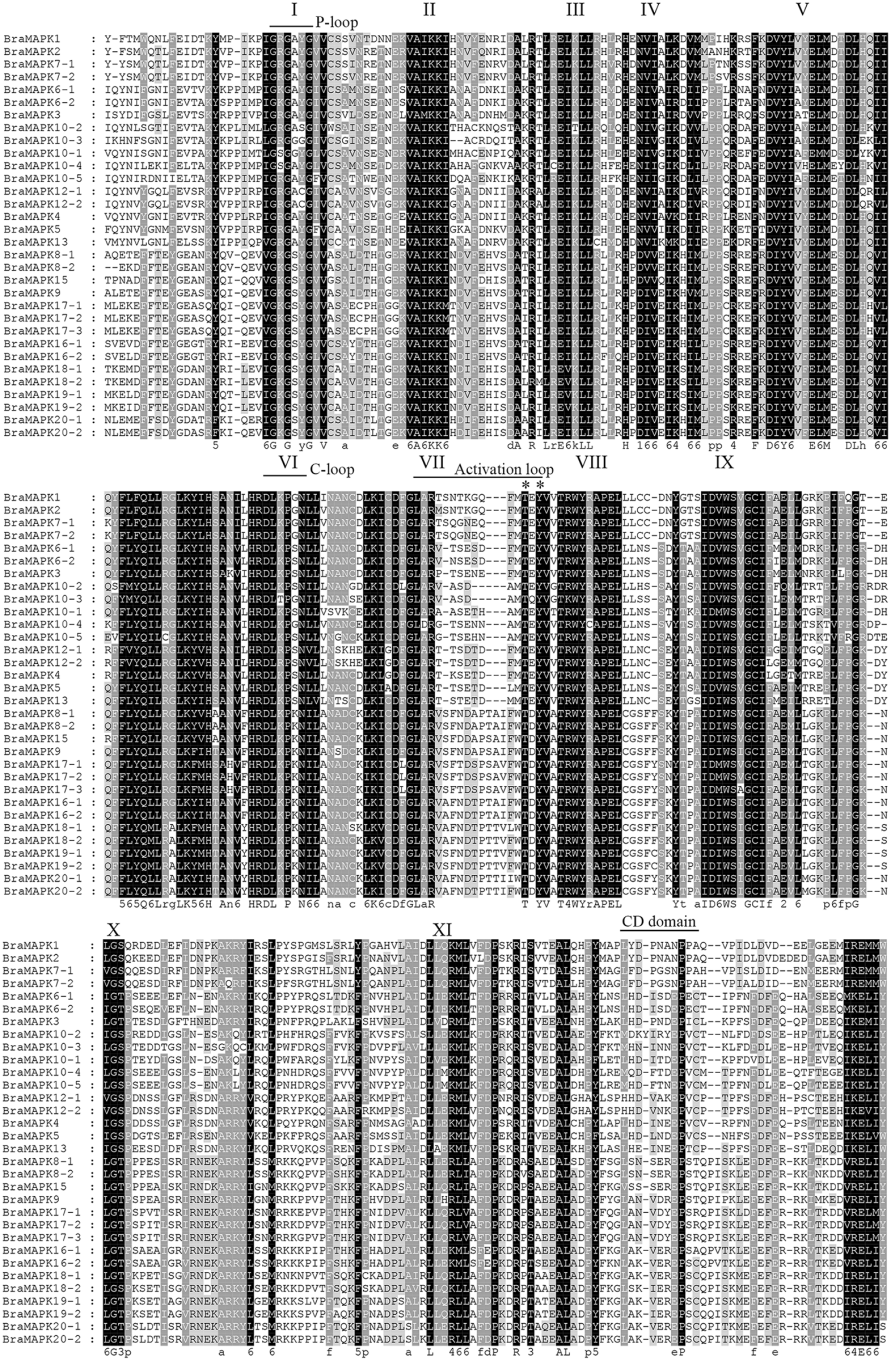
Multiple sequence alignment of the kinase domains of 32 BraMAPK proteins. Alignment was performed using ClustalW2 and displayed using GeneDoc. Identical sequences are highlighted in black and similar residues in gray shading. The 11 kinase subdomains are in italicized roman numerals (I to XI) above the sequence. P-loop, C-loop, activation-loop motifs and common docking domain are indicated by lines above the alignments. The phosphorylation-activation motif TXY is indicated by an asterisk. Bra: *B*. *rapa*.

In plants, MAPKs were divided into four groups (A-D) based on the presence of the TEY or TDY motif in their phosphorylation sites [6]. TEY MAPKs contain A, B and C groups, whereas group D compromises TDY MAPKs. To classify BraMAPK proteins and determine the evolutionary relationship of MAPK families in *Arabidopsis* and *B*. *rapa*, we firstly constructed a smaller NJ tree based only on the multiple alignment of 20 AtMAPKs and 32 BraMAPKs, using *Arabidopsis* CASEIN KINASE II protein (AtCKA2) as an outgroup. Based on the phylogenetic analysis, the BraMAPK proteins were divided into four groups (Fig 1), with each group containing at least four BraMAPK proteins corresponding to at least three MAPK homologues in *Arabidopsis*. BraMAPKs belonging to groups A, B, and C all possess a TEY motif, except for BraMAPK10-3, which harbors a TQY motif, whereas those of group D possess a TDY motif at the activation site (Fig 3 and Table 1). Despite the very recent duplication events, no significant expansion was observed for BraMAPKs in groups B and C, since the gene number in the whole genome was the same for *Arabidopsis* and *B*. *rapa*, and there were 5 and 4 BraMAPKs in groups B and C, respectively (Fig 1 and S3 Table). Furthermore, we found that there were no orthologs of AtMAPK4 and AtMAPK11 in *B*. *rapa*. Furthermore, BraMAPKs in groups A and D experienced a significant expansion, as 8 and 15 BraMAPKs were present in *B*. *rapa*, while 3 and 8 corresponding orthologs existed in *Arabidopsis*. It is noteworthy that there were five copies of *BraMAPK10* genes in *B*. *rapa*, which might be the result of genome-wide duplication and subsequent partial tandem duplication events.

To further investigate the evolutionary relationship between BraMAPKs and MAPK proteins from other plants, we constructed a second, larger NJ tree based on the amino acid sequence alignments of 91 MAPK proteins from *Arabidopsis*, *B*. *rapa*, rice, and maize, and 17 well-known plant MAPK proteins (Fig 2). The phylogenetic tree showed that other plant MAPK proteins also fell into four groups. Group D was the largest clade and contained 49 plant MAPK proteins, while the smallest clade was group C, which only contained 15 MAPKs. Furthermore, groups A and B possessed 27 and 17 MAPKs, respectively. Interestingly, we found that group D is the largest group of MAPKs in most plant species, and the number of group D MAPKs in *B*. *rapa* genome is significantly higher than that of other species.

As shown in Fig 2, phylogenetic relationship of plant MAPK proteins was consistent with the phylogeny of plant species. MAKP members from *Arabidopsis* and *B*. *rapa* first formed a Brassicaceae branch, which further clustered with other well-known MAPK proteins to form a distinct dicot MAPK clade. For example, AtMAPK3 and BraMAPK3 first formed the Brassicaceae MAPK3 branch, and then both further clustered with *Catharanthus roseus* MPK3 (CrMPK3) and NtWIPK from tobacco to form the clade of dicot MAPK3 proteins, while TaWCK-1, OsMAPK5, ZmMAPK3-1, and ZmMAPK3-1 clustered together and formed the monocot clade of MAPK3 proteins. The Brassicaceae-specific duplication events in group D before *Brassica*-specific genome triplication could be found on the phylogenetic tree (Fig 2). For example, the evolutionary relationship among MAPK8, MAPK9, and MAPK15 in *Arabidopsis* and *B*. *rapa* is different from that among the related homologues in rice and maize (ZmMPK8, ZmMPK15, OsMPK16, and OsMPK17). Similar Brassicaceae-specific duplication events also occurred in some clades of group B (MAPK4 and MAPK11) and C (MAPK1 and MAPK2), but not for group A, as the two MAPKs (MAPK3 and MAPK6) in this group formed two separate clades, and then gathered together, indicating that the ancestral gene of MAPK3 and MAPK6 in group A already existed after the divergence of the four MAPK groups, but before the monocot—eudicot divergence. In general, MAPK proteins underwent at least two divergence events in the plant kingdom. The first divergence occurred before the monocot-dicot split and formed the ancestral genes for each of the groups in the common ancestor of monocot and dicot plants, while the second divergence happened concurrently with the divergence of monocots and dicots, causing the formation of monocot- and dicot-specific ancestral *MAPK* genes.

To validate the two NJ trees, we constructed the corresponding ML trees using the same alignment of MAPK proteins through PhyML version 3.0.1 (S1 and S2 Figs). The ML tree topologies of plant MAPK proteins coincided with the corresponding NJ trees, indicating that both the inferred NJ and ML trees accurately reflected the evolutionary process underlying the development of plant MAPK proteins.

### Chromosomal distribution and genomic structure analyses

**Fig 4:**
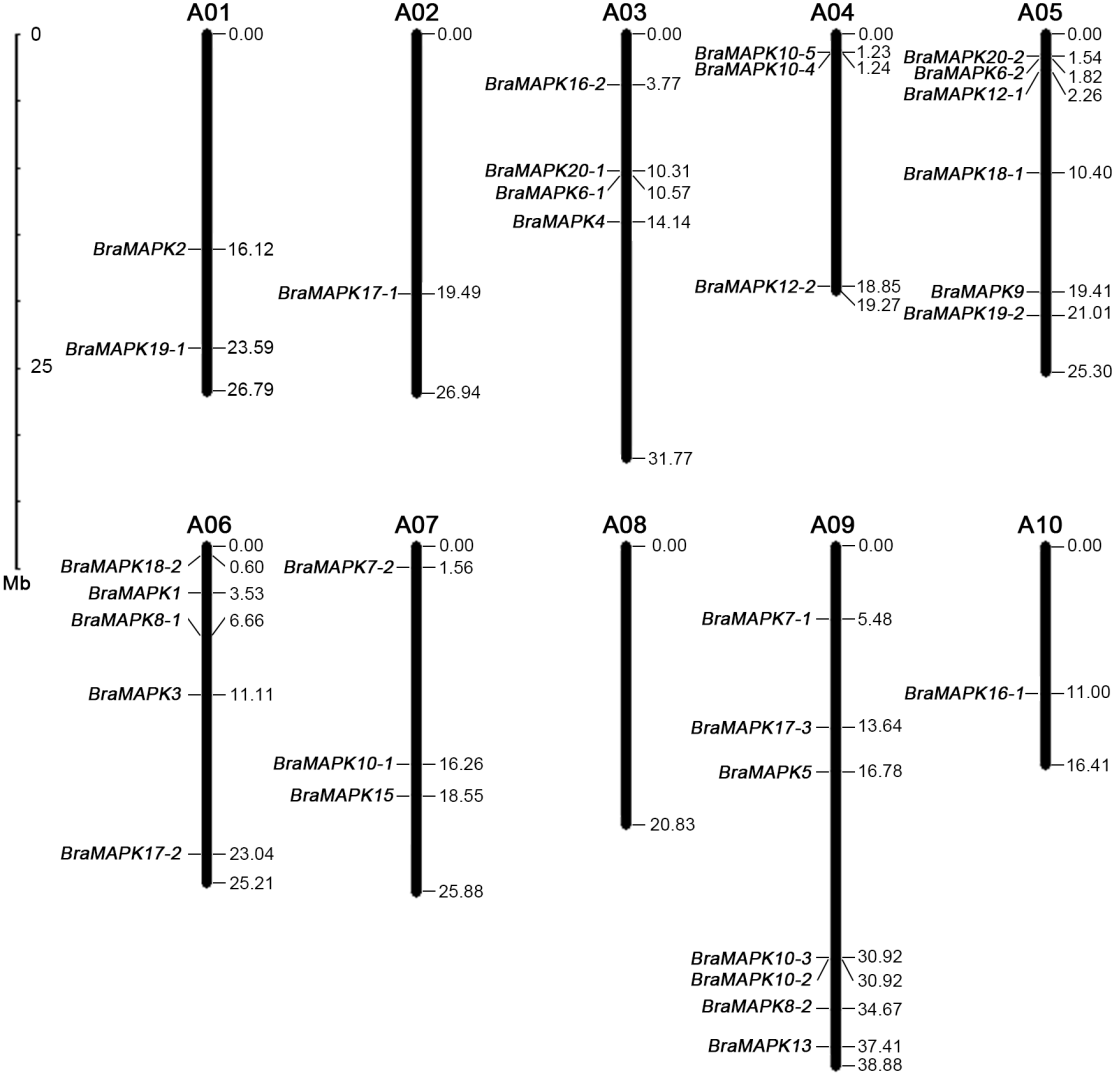
Chromosomal locations of 32 *BraMAPK* genes. The scale on the left is in megabases. The chromosome numbers are indicated above each chromosome. The gene names are on the left side of each chromosome, according to the approximate physical location (right side) of each *BraMAPK* gene.

To determine the chromosomal distribution of the identified *BraMAPKs*, we subjected the genomic sequences of 32 *BraMAPK* genes to BLASTN analysis against the 10 *B*. *rapa* chromosomes. As shown on the location image (Fig 4), the 32 *BraMAPK* genes were successfully mapped to 9 of the 10 chromosomes present in the genome. No *BraMAPK* genes were located on chromosome 8, and only one *BraMAPK* was found on chromosomes 2 and 10. By contrast, more than half of the *BraMAPK* members were distributed on chromosomes 5, 6, and 9. Two pairs of tandem duplicated genes, *BraMAPK4/5* and *BraMAPK2/3*, occurred on chromosomes 4 and 9, respectively.

Genomic organization can provide important insight into the evolution of a gene family. To determine the exon-intron structure of *AtMAPK* and *BraMAPK* genes, we aligned and compared the CDSs and their corresponding genomic sequences. We found that different groups of *MAPK* genes have strikingly different exon-intron structures, but that the structures of *MAPK* members in the same group were highly conserved in *Arabidopsis* and *B*. *rapa* (Fig 1). The *AtMAPK* and *BraMAPK* members in Group A and B were composed of six exons and five introns, while those of Group C all had two exons and one intron. Eight to eleven exons were present in the *AtMAPK* and *BraMAPK* genes in Group D. The length of some introns differed between *AtMAPKs* and their orthologs in *B*. *rapa* (Fig 1). The first introns of *BraMAPK10-2* and *BraMAPK10-3* were longer than those of *AtMAPK10* and other members of the *BraMAPK10* gene family. The first introns of *BraMAPK1*, *BraMAPK7-1*, *BraMAPK7-2*, and *BraMAPK8-1*, and the third intron of *BraMAPK13* were also longer than those of the corresponding orthologous *MAPK* genes in *Arabidopsis*, while in rare cases the converse was true. For instance, the first intron of *AtMAPK9* was longer than that of *BraMAPK9*.

### Conserved domain analysis of BraMAPKs

In previous studies, MAPKs from several plant species were characterized based on the presence of characteristic features of serine/threonine protein kinases, namely, the 11 conserved MAPK subdomains and the phosphorylation-activation TXY motifs [2]. The amino acid sequence alignment of the 32 BraMAPKs identified here is shown in Fig 3. All of these MAPKs contained the 11 conserved kinase subdomains and a TEY or TDY motif in the activation loop between the kinase catalytic subdomains VII and VIII. Furthermore, the N-terminal regions of the 32 BraMAPKs have a glycine-rich motif (GRG[A/S]YG) located in the phosphate-binding loop (P-loop) in subdomain I, which acts as an ATP- and GTP-binding site [74] (Fig 3). The conserved catalytic loop (C-loop, HRDLKP[G/S/K]N), which harbors an Asp (D) residue that is the key site of the phosphorylation reaction, was also present in subdomain XI of BraMAPKs (Fig 3).

To gain insight into the structural diversity of the *AtMAPK* and *BraMAPK* genes, we submitted the deduced protein sequences to the online MEME/MAST system to search for conserved motifs [65]. A total of 31 motifs with e-values of < 1e-10 were identified amongst the 52 AtMAPK and BraMAPK proteins using the MEME program and uploaded into MAST for motif detection (S3 Fig and S4 Table). We found that motif 1 (113 aa) and motif 5 (29 aa) were present in all input AtMAPK and BraMAPK proteins, and that 18 motifs only existed in group D MAPK proteins, among which was motif 2 (113 aa), which possessed the conserved TDY motif (TDYVATRWYRAPEL) in the activation loop. Three motifs (motifs 4, 6, and 15) were present in group A, B, and C MAPKs in *Arabidopsis* and *B*. *rapa*, and one of these, motif 4 (57 aa), contained the TEY signature motif (TEYVVTRWYRAPEL) (S4 Table).

The MAPK common docking (CD) domain consists of two negatively charged amino acid residues and functions as a docking site for MAPK kinases, substrates, and negative regulators [75]. This conserved domain was observed in the extended C-terminal region of the BraMAPK members belonging to the A and B groups (BraMAPK3, 4, 5, 6–1, 6–2, 10–1, 10–4, and 13), indicating that the acidic cluster (LH[D/E]XX[D/E]EPXC) may establish electrostatic interactions with upstream factors and form a modular recognition system that mediates connectivity (Fig 3). The BraMAPK members of group D did not possess the CD domain. It has been reported that the AtMPK proteins in group C contain a modified CD domain [75], but the modified CD domain (VPIDLDVDE[D/E]LXXE) was only observed in BraMAPK1 and BraMAPK2. The group distribution of the CD domain for BraMAPK proteins is in accordance with previous reports for MAPK proteins in maize and grapevine [40,43].

### Tissue-specific expression patterns of *BraMAPK* genes

**Fig 5:**
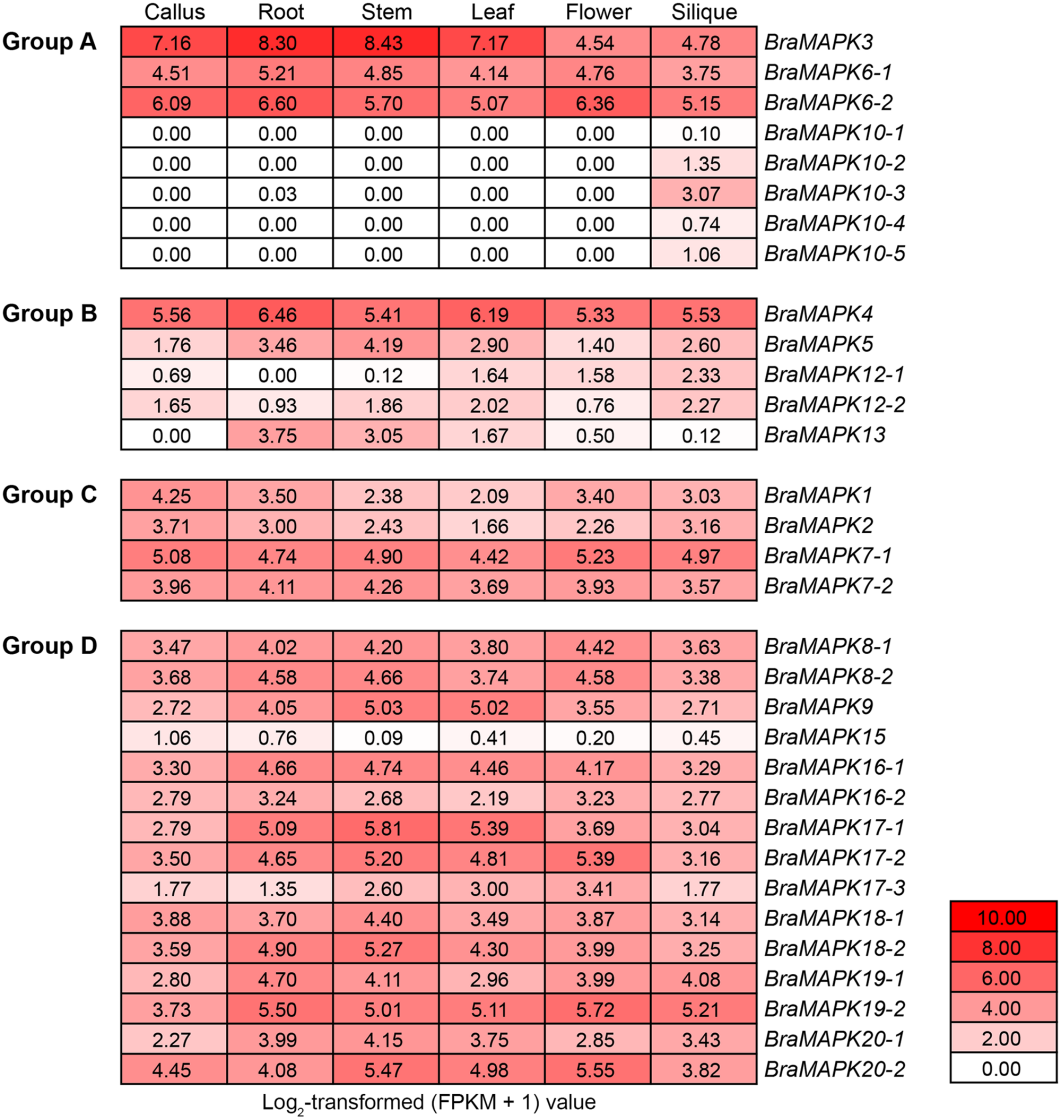
Tissue-specific expression profiles of *BraMPK* genes. The tissue-specific expression levels of 32 *BraMPK* genes were obtained from the RNA-seq data (accession number GSE43245) and resulting FPKM values. This heat map was generated based on the log_2_-transformed (FPKM + 1) values of 32 *BraMAPK* genes in six different tissues (callus, root, stem, leaf, flower, and silique). Red indicates high expression and white indicates low expression.

The tissue specificity of the 32 *BraMAPK* genes was investigated using available RNA-seq data and the resulting FPKM values from six different tissues [66,67]. Based on the log_2_-transformed (FPKM + 1) values, expression levels of *BraMAPK* genes were classified as low (≤2), moderate (>2 and ≤6), and high (>6). We found that the expression levels of the 32 *BraMAPKs* varied considerably (Fig 5). In groups A, B and C, *BraMAPK3*, *BraMAPK4* and *BraMAPK7-1* were expressed at relatively higher levels than other members within the same group, respectively. Except for *BraMAPK15*, the rest *BraMAPK* genes in group D all showed moderate expression in at least three tissues.

Among the 32 *BraMAPK* genes, five *BraMAPK* genes (i.e., *BraMAPK10-1*, *10–2*, *10–4*, *10–5* and *15*) were expressed at low levels in all tested tissues. *BraMAPK3* showed the highest expression in callus, root, stem and leaf, whereas *BraMAPK4* and *6–2* possessed the highest transcription levels in flower and silique, respectively. Five members of *BraMAPK10* gene subfamily were all unique to silique. Interestingly, the duplicated *BraMAPK* gene pairs (i.e., genes in the *BraMAPK6*, *7*, *16*, *17* and *19* subfamilies) showed similar distributions, but significant differences in transcript abundance (Fig 5 and S5 Table). For example, duplicated members from the *BraMAPK6* subfamily showed similar expression patterns, but the level of *BraMAPK6-2* transcript was significantly higher than that of *BraMAPK6-1* (*p*-value = 0.0002).

### Expression patterns of *BraMAPK* genes under abiotic stresses

**Fig 6:**
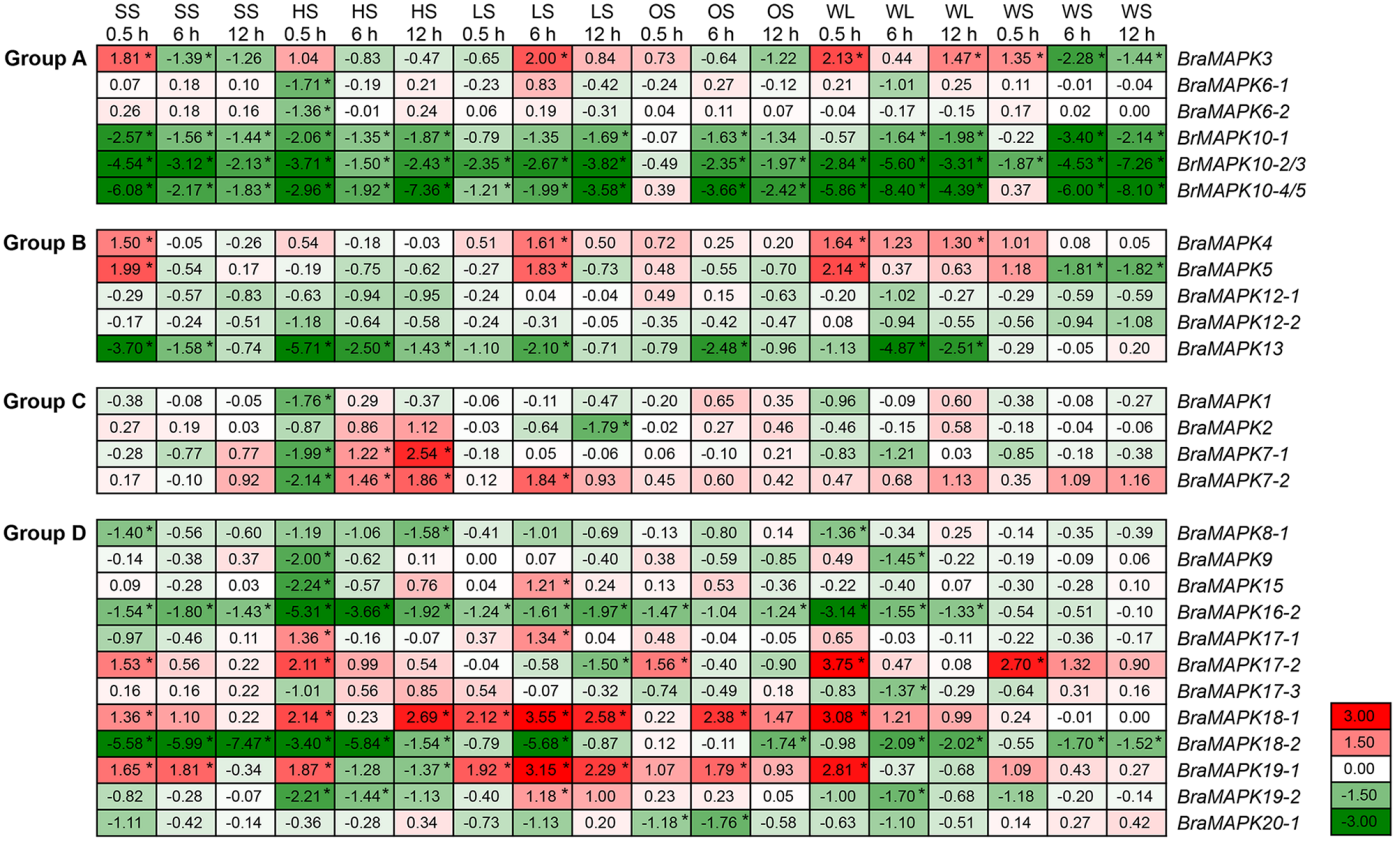
Heat map showing expression levels of *BraMPK* genes in the seedling leaves under abiotic stresses. Transcription levels of *BraMPAK* genes were determined by qRT-PCR with gene-specific primers under salt (200 mM NaCl), heat (37°C), cold (4°C), osmotic (10% PEG-8000), waterlogging, and wound stresses. The relative expressions (ΔΔCt) were compared to expressions in mock-treated samples, and used to create the heat map. Data represents a mean value of three repeats from three independent qRT-PCR assays. SS, HS, LS, OS, WL, and WS denote salt, heat, cold, osmotic, waterlogging, and wound stress treatments, respectively. Asterisk (*) on the right corner of number indicate the significant difference (*p*-value < 0.05) compared with mock-treated controls. The color scale represents the relative expression levels (red refers to up-regulation of gene expression, green to down-regulation of gene expression, and white indicates unchanged gene expression).

To investigate the contribution of *BraMAPK* to various abiotic stress responses, we subjected 4-week-old *B*. *rapa* seedlings to different abiotic stresses and monitored the expression patterns of 27 *BraMAPK* genes using real-time PCR. All of the tested *BraMAPK* genes displayed altered expression patterns, either induction or suppression, in response to at least one kind of abiotic stress treatment, with the exception of *BraMAPK8-2*, *BraMAPK16-1*, and *BraMAPK20-2*, which had transcript abundances that were too weak to detect (Fig 6 and S4 Fig). The expression of five *BraMAPKs* (*BraMAPK3*, *4*, *5*, *17–2*, and *18–1*) was induced after 0.5 h of salt stress, but declined after 6 h and 12 h of treatment. *BraMAPK10-1*, *BraMAPK10-2/3*, *BraMAPK10-4/5*, *BraMAPK13*, *BraMAPK16-2*, and *BraMAPK18-2* were suppressed by salt stress throughout the 12-h treatment. After heat treatment, *BraMAPK3*, *BraMAPK4*, *BraMAPK17-1*, *BraMAPK17-2* and *BraMAPK19-1* were rapidly up-regulated at the 0.5-h time-point, but declined at 6 h and 12 h, and *BraMAPK2*, *BraMAPK7-1* and *BraMAPK7-2* were up-regulated only after 6 h and 12 h of heat stress treatment. In response to chilling stress, *BraMAPK18-1* and *BraMAP19-1* were up-regulated throughout the treatment process, and *BraMAPK3*, *BraMAPK4*, *BraMAPK7-2*, *BraMAPK15*, *BraMAPK17-1* and *BraMAPK19-2* also displayed slight elevations in expression after 6 h of treatment. At 6 h of osmotic stress treatment, the expression of *BraMAPK18-1* (*p*-value = 0.0194) and *BraMAPK19-1* (*p*-value = 0.0130) was significantly up-regulated, whereas that of *BraMAPK10-1*, *BraMAPK10-2/3*, *BraMAPK10-4/5*, *BraMAPK13* and *BraMAPK20-1* were significantly down-regulated. Waterlogging treatment caused induction of *BraMAPK3*, *BraMAPK4*, *BraMAPK*5, *BraMAPK17-2*, *BraMAPK18-1*, and *BraMAPK19-1* at 0.5 h of treatment, followed by rapid decline at 6 h and 12 h of treatment. Similar trends were observed when the *BraMAPK17-2* was subjected to wound stress (Fig 6 and S4 Fig).

### Expression profiles of *BraMAPK* genes in response to hormone treatments

**Fig 7:**
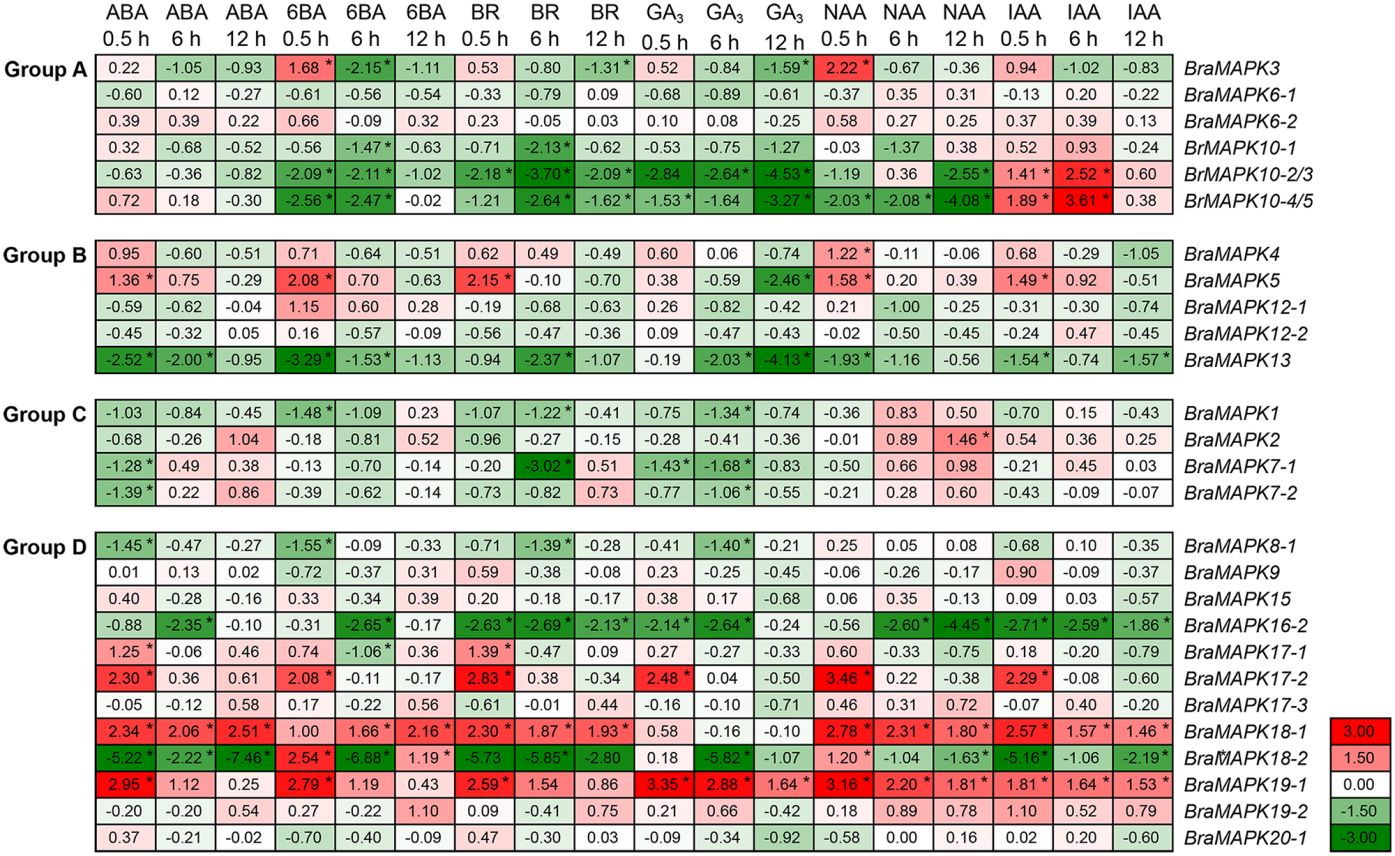
Expression profiles of *BraMPK* genes in the seedling leaves treated with different hormones. Expression levels of *BraMAPK* genes assayed by qRT-PCR under ABA (100 μM), 6-BA (10 μM), BR (2.5 μM), GA_3_ (25 μM), NAA (10 μM) and IAA (10 μM) hormone treatments. The relative expressions (ΔΔCt) were compared to expressions in mock-treated samples, and used to create the heat map. Data represents a mean value of three repeats from three independent qRT-PCR assays. Asterisk (*) on the right corner of number indicate the significant difference (*p*-value < 0.05) compared with mock-treated controls. The color scale represents the relative expression levels (red refers to up-regulation of gene expression, green to down-regulation of gene expression, and white indicates unchanged gene expression). ABA: abscisic acid; 6-BA: 6-benzyladenine; BR: brassinolide; GA_3_: gibberellic acid; NAA: *α*-naphthaleneacetic acid; IAA: indole acetic acid.

We also investigated the expression of *BraMAPK* genes in response to different hormone treatments (Fig 7 and S5 Fig). The transcript levels of *BraMAPK18-1* and *BraMAPK19-1* were up-regulated in response to all of the treatments, whereas those of *BraMAPK13* and *BraMAPK16-2* were down-regulated following all of the treatments. *BraMAPK*5, *BraMAPK17-1*, *BraMAPK17-2*, *BraMAPK18-1* and *BraMAPK19-1* were all significantly induced at 0.5 h of ABA treatment, and only *BraMAPK18-1* was sustainedly activated after 6 h and 12 h of ABA treatments. Furthermore, *BraMAPK3*, *BraMAPK*5, *BraMAPK17-2*, *BraMAPK18-2* and *BraMAPK19-1* were induced following a short-term treatment (0.5 h) with 6-BA, but were declined after longer treatments (6 and 12 h; Fig 7 and S5 Fig). Short-term treatments with BR and the auxin (NAA and IAA) induced *BraMAPK17-2* expression, but transcript declined to basal levels after 6 h of treatment. Interestingly, *BraMAPK10-2/3* and *BraMAPK10-4/5* seemed to be specifically induced after 0.5 h and 6 h of IAA treatment, suggesting the importance of these genes in the IAA signaling pathway. Hormone treatments had diverse effects on the expression patterns of members of the *BraMAPK17*, *BraMAPK18*, and *BraMAPK19* gene families. In these three gene families, *BraMAPK17*-2, *BraMAPK18-1*, and *BraMAPK19-1* (*p*-value = 0.0002, 0.0000 and 0.0000, respectively) always showed significantly higher induction and abiotic stress treatments than other members in the same gene subfamily (S5 Table). However, members of the *BraMAPK12* (*p*-value = 0.4504) gene subfamily did not show differences in expression patterns after the hormone treatments (Fig 7 and S5 Fig).

Pearson correlation coefficient of relative expression levels of all *BraMAPK* genes between qRT-PCR and RNA-seq methods is 0.8954 (S6 Table), a strong positive correlation (S6 Table), indicating that the results obtained from two methods are consistent. The expression patterns of *AtMAPK* genes showed relatively high consistency to *BraMAPK* genes, since the coefficients of relative expression levels of *AtMAPK* genes in leaves are 0.6600 and 0.6727 to those of orthologs in *B*. *rapa* derived from qRT-PCR and RNA-seq methods, respectively (S6 Table).

## Discussion

Extensive manual checks to original sequences, especially obtained from a non-model genome, are necessary for further analyses of gene families. For the model plants *Arabidopsis* and rice, extensive manual curation has improved the accuracy of annotations and thereby facilitated the development of accurate biological databases [55] and paved the way for further experimental or computational studies [76]. In this study, we identified 32 *MAPK* genes in the *B*. *rapa* genome. However, 13 of the 32 original *BraMAPK* genes identified in BRAD were found to be inaccurate, due to different types of errors. Using the 32 original *BraMAPK* sequences as is would have resulted in inaccurate gene structure annotations and faulty phylogenetic reconstruction of the *BraMAPK* gene family. Hence, it is highly recommended to manually curate gene models of *B*. *rapa* before investigating the evolutionary features, transcriptional characteristics, and putative functions of important gene families.

In *B*. *rapa*, the number of *BraMAPK* genes is higher than those in other plant species reported to date. Previously studies revealed that the current genome of *B*. *rapa* was shaped by whole-genome triplication (WGT) followed by extensive diploidization [77], which might be the principal reason for expansion of the *MAPK* gene family in *B*. *rapa*. Compared with ten *AtMAPK* genes, ten corresponding *BraMAPK* subfamilies in the triplication blocks kept at least two subfamily members, providing an evidence that WGT led to expansion of the *MAPK* gene family in *B*. *rapa*, though gene loss occurred after genome triplication. Tandem duplication (TD) is another important way for gene expansion in plants. A total of 2,137, 1,569, 1,751, and 1,135 tandem gene arrays were detected in *B*. *rapa*, *A*. *thaliana*, *Arabidopsis lyrata*, *Thellungiella parvula*, respectively [78]. We found that TD also made an important contribution to the size of *BraMAPK* gene family. Five members of the *BraMAPK10* subfamily are located in triplication block N, one of the 24 conserved genomic blocks in the recently sequenced genomes of Brassicaceae species [51]. Two pairs of orthologous genes, *BraMAPK10-2/3* and *BraMAPK10-4/5*, both originated from tandem duplication events after genome triplication. Similar with plant *Auxin*/*indoleacetic acid* (*Aux*/*IAA*) gene family that group diversification of the *IAA* family occurred pre-dated the monocot/dicot divergence [79], the divergence of MAPK proteins between monocot and dicot plants was readily observed in each group. A comparison of the number of *MAPKs* in different plant genomes showed that each group contained at least two MAPKs per species, suggesting that the plant *MAPK* gene family is an ancient family, and that diversification of the four groups occurred before the ancient monocot—eudicot divergence.

Tissue specific detection of *BraMAPK* genes showed that the expression pattern of each member of a duplicated *BraMAPK* paralogous pair differed from that of the other, indicating that sub-functionalization of genes duplicated by polyploidy happened occasionally in *B*. *rapa*. A similar phenomenon was observed in a genome-wide analysis of the *pectate lyase-like* (*PLL*) genes in *B*. *rapa*, which revealed the existence of three *PLL* gene subfamilies in plants, among which subfamily II might have evolved via gene neo-functionalization or sub-functionalization [80]. As an example of sub-functionalization, two members of the *BraMAPK6* subfamily are expressed in all the tested tissues, but the expression level of one member (*BraMAPK6-2*) is significantly higher than that of the other (*BraMAPK6-1*) (S5 Table). Many current models consider that gene loss occurs rapidly after whole genome duplication, and functional divergence (either by sub-functionalization or neo-functionalization) can explain why the duplicated genes escape extinction [78]. It could be speculated that retention of large number of duplicated *BraMAPK* paralogous might be caused by sub-functionalization in *B*. *rapa*.

Recently, many studies have focused on *MAPK* genes in groups A and B and the genes in these two groups have been widely studied [2]. In *Arabidopsis*, AtMAPK3 and AtMAPK6 of group A are the most prominent kinases, and have all been strongly associated with numerous abiotic and biotic stresses [10]. *AtMAPK3* was induced transcriptionally by various environmental stresses such as mechanical stimuli, low temperature, and osmotic stress [81]. In this study, *BraMAPK3* showed a rapid response to salt, heat, waterlogging, wound, 6-BA and NAA treatments, which indicated that *BraMAPK3* might be an important regulator in response to abiotic stresses. After low-temperature, low-humidity, touch and wounding treatments, the transcript of *AtMAPK6* remained unchanged, whereas the kinase activity of AtMAPK6 was activated rapidly [82]. It is notable that little or no stress-induced gene expression of *BraMAPK6-1* and *BraMAPK6-2* was observed in any of the treatments, suggesting that activation of BraMAPK6 protein kinase activities might be not correlated with their transcript levels, similar with AtMAPK6. As the biggest *BraMAPK* gene subfamily, five *BraMAPK10* members were originated from both WGT and TD. The expression of *BraMAPK10* gene family members was repressed by most hormone treatments, similar to their response to abiotic stresses, except for IAA treatment. Since the *BraMAPK10* genes were specifically expressed in the silique and induced by IAA, we speculate that the 5 members of the *BraMAPK10* subfamily are key regulators of silique growth and development, and associated with abiotic stress and hormone signal transduction pathways as negative regulators. Group B MAPKs, represented by *Arabidopsis* MAPK4 and MAPK11, are implicated in pathogen defense and abiotic stress responses [83]. *BraMAPK4* and *BraMAPK5* from group B were induced by salt, cold, wound, waterlogging and ABA treatments, suggesting the involvement of these genes in abiotic stress tolerance and ABA signal transduction in *B*. *rapa*. *Arabidopsis* MAPK13 can be activated by AtMAPKK6, and this module is required for initiating and/or sustaining pericycle cell division during lateral root initiation in *Arabidopsis* [84]. The expression of *BraMAPK13* was repressed by most of stresses and hormone treatments, suggesting that *BraMAPK13* may function in an early stage of stress signaling transduction as negative regulators in *B*. *rapa*. Recently, all group C MPAKs in *Arabidopsis*, AtMAPK1, AtMAPK2, AtMAPK7 and AtMAPK14, are activated by ABA in an MKK3-dependent manner, providing evidence for a role of an ABA-induced MAPK pathway in plant stress signaling [85]. In this study, only *BraMAPK1* and two members of *BraMAPK7* showed rapid response to ABA treatment, and their expression was repressed at 0.5 h of treatment, which suggested that these *MAPK* genes might also have important functions in ABA signaling. *MAPK* genes of group D have not been as well studied as those of group A, but previous studies have shown that *Arabidopsis* MAPK9 was found to be highly and preferentially expressed in guard cells, and positively regulate reactive oxygen species (ROS)-mediated ABA signaling [86]. However, transcription level of *BraMAPK9* was not detected obviously induced by ABA and other treatments, which suggested that the involvement of *BraMAPK9* in ABA signaling might be regulated at the level of translation. It has reported that the induction by osmotic stress of the *Arabidopsis MAPK17* and its homolog in maize suggests that *MAPK17* and its ortholog could be good candidates for further functional characterizations and involvement in abiotic stress adaptation [87]. It is interesting that expression of *BraMAPK17-2* was induced by most of stress and hormone treatments, and the hormone induction levels of *BraMAPK17-2* was at least two-fold higher than that of *BraMAPK17-1* at early stage of treatments. These results suggested that *BraMAPK17* genes might have similar functions with *AtMAPK17*, and involved in response to various hormone signaling. It is notable that *BraMAPK19-1* was induced by all hormone treatments, and that *BraMAPK18-1* was induced by all hormones except GA_3_. Considering the induction level of *BraMAPK18-1* and *BraMAPK19-1* expression after abiotic and hormone treatments, these two *BraMAPK* genes might be the main kinases in group D at the level of transcription, and they might be involved in responses to diverse hormone signaling pathways and the crosstalk amongst these pathways, and have important functions in plant resistance and growth regulation.

In summary, our study provides a comprehensive overview of the *MAPK* gene family in *B*. *rapa*, and demonstrates that *BraMAPK* plays vital roles in response to abiotic stresses and hormone signal transduction. These results provide an accurate reference of *BraMAPK* genes, which lays a solid foundation for further characterization of the physiological and biochemical functions of *BraMAPKs*.

## Supporting Information

S1 Table Primers used for sequence correction of *BraMAPK* genes. (XLSX)

S2 Table Primers used for qRT-PCR detection of *BraMAPK* genes. (XLSX)

S3 Table Comparison of number of MAPKs in different plant genomes. (DOCX)

S4 Table Predicted motifs of AtMAPK and BraMAPK proteins. (DOCX)

S5 Table ANOVA analysis of sub-functionalization of duplicated members in the same *BraMAPK* gene subfamily. (XLSX)

S6 Table Comparison of expression levels of MAPK genes in leaves between *B*. *rapa* and *A*. *thaliana*. (XLSX)

S1 Fig ML Phylogenetic tree of *MAPK* genes in *Arabidopsis* and *B*. *rapa*. The phylogenetic tree was generated by the PhyML version 3.0.1 from alignment of amino acid sequences of 20 AtMAPKs, 32 BrMAPKs and AtCKA2, using the JTT model of amino acid substitution, an estimated gamma distribution parameter, and 100 bootstrap replicates. The tree was displayed with FigTree v1.4.0. At: *Arabidopsis thaliana*; Bra: *B*. *rapa*.
(TIF)

S2 Fig ML Phylogenetic tree of plant *MAPK* proteins. The phylogenetic tree derived by the ML method with bootstrap analysis (100 replicates) from alignment of amino acid sequences of MAPK proteins in *Arabidopsis*, *B*. *rapa*, rice, *Zea mays*, and other plants using PhyML version 3.0.1 program. The tree was displayed with FigTree v1.4.0. Only bootstrap values greater than 50% are denoted at the nodes. At: *A*. *thaliana*; Bra: *B*. *rapa*; Cr: *C*. *roseus*; Gh: *G*. *hirsutum*; Md: *M*. *domestica*; Nt: *N*. *tabacum*; Os: *O*. *sativa*; Ps: *P*. *sativum*; St: *S*. *tuberosum*; Ta: *T*. *aestivum*; Zm: *Z*. *mays*.
(TIF)

S3 Fig Conserved motif analysis of MAPK proteins in *Arabidopsis* and *B*. *rapa*. The phylogenetic tree (left panel) was generated from an amino acid sequence alignment of 20 AtMAPKs and 32 BrMAPKs using MEGA 6.0 with the ClustalW2 program and the NJ method (1000 bootstrap replicates), and displayed using FigTree v1.4.0. Only bootstrap values greater than 50% are denoted at the nodes. The 52 MAPK proteins in *A*. *thaliana* and *B*. *rapa* were clustered into four distinct groups (Groups A, B, C, and D). Distribution of conserved motif in AtMAPKs and BrMAPKs is shown in the right panel. A total of 31 motifs with an e-value of < 1e-10 were identified using the MEME program. Each motif is represented by a colored box. Box length corresponds to motif length. The specific lengths, groups, and e-values of each motif are listed in S3 Table. At: *A*. *thaliana*; Bra: *B*. *rapa*.
(TIF)

S4 Fig Expression patterns of *BraMPK* genes in the seedling leaves under abiotic stresses. Transcription levels of *BraMPAK* genes were determined by qRT-PCR with gene-specific primers under salt (200 mM NaCl), heat (37°C), cold (4°C), osmotic (10% PEG-8000), waterlogging, and wound stresses. The expression levels were normalized against *BraUBC21* and *BraGAPDH* genes using 2^–ΔΔCT^ method [69]. Data are means ± SD (*n* = 3) and are representative of similar results from three independent experiments. Asterisk (*) on top of error bar indicate the significant difference (*p*-value < 0.05) compared with mock-treated controls. SS, HS, LS, OS, WL, and WS denote salt, heat, cold, osmotic, waterlogging, and wound stress treatments, respectively.
(TIF)

S5 Fig Expression profiles of *BraMPK* genes in the seedling leaves treated with different hormones. Expression levels of *BraMAPK* genes assayed by qRT-PCR under ABA (100 μM), 6-BA (10 μM), BR (2.5 μM), GA_3_ (25 μM), NAA (10 μM) and IAA (10 μM) hormone treatments. The expression levels were normalized against *BraUBC21* and *BraGAPDH* genes using 2^–ΔΔCT^ method [69]. Data are means ± SD (*n* = 3) and are representative of similar results from three independent experiments. Asterisk (*) on top of error bar indicate the significant difference (*p*-value < 0.05) compared with mock-treated controls. ABA: abscisic acid; 6-BA: 6-benzyladenine; BR: brassinolide; GA_3_: gibberellic acid; NAA: *α*-naphthaleneacetic acid; IAA: indole acetic acid.
(TIF)

S1 File Final corrected sequences of 13 *BraMAPK* genes in *B*. *rapa*. (TXT)
