# Supplementary material for: Genome-Wide Survey and Expression Profile Analysis of the Mitogen-Activated Protein Kinase (MAPK) Gene Family in Brassica rapa
Source: PLoS One. 2015 Jul 14;10(7):e0132051. doi: 10.1371/journal.pone.0132051 (PMC4501733; doi:10.1371/journal.pone.0132051)
Supplement: S3 Table — (DOCX) [file pone.0132051.s003.docx]

**Table S3. Comparison of number of MAPKs in different plant genomes.**

| Group | *A. thaliana*^[3]^ | *B. rapa* | *P. trichocarpa*^[45]^ | *V. vinifera*^[40]^ | *M. acuminata*^[46]^ | *S. lycopersicum*^[42]^ | *O. sativa*^[36, 37]^ | *B. distachyon*^[38]^ | *Z. mays*^[43]^ | *^a^N. tabacum*^[44]^ | *^a^M. morus*^[39]^ |
| --- | --- | --- | --- | --- | --- | --- | --- | --- | --- | --- | --- |
| A | 3 | 8 | 4 | 2 | 5 | 3 | 2 | 2 | 4 | 3 | 2 |
| B | 5 | 5 | 4 | 3 | 5 | 4 | 2 | 2 | 2 | 4 | 3 |
| C | 4 | 4 | 4 | 2 | 2 | 2 | 2 | 3 | 2 | 2 | 2 |
| D | 8 | 15 | 9 | 5 | 10 | 7 | 11 | 9 | 11 | 2 | 1 |
| E |  |  |  |  |  |  |  |  |  | 4 | 2 |
| F |  |  |  |  |  |  |  |  |  | 2 |  |
| Total | 20 | 32 | 21 | 12 | 22 | 16 | 17 | 16 | 19 | 17 | 10 |

a: *MAPK* genes were divided into six groups in these plant species
