# Supplementary material for: Genome-Wide Survey and Expression Profile Analysis of the Mitogen-Activated Protein Kinase (MAPK) Gene Family in Brassica rapa
Source: PLoS One. 2015 Jul 14;10(7):e0132051. doi: 10.1371/journal.pone.0132051 (PMC4501733; doi:10.1371/journal.pone.0132051)
Supplement: S4 Table — (DOCX) [file pone.0132051.s004.docx]

**Table S4. Predicted motifs of AtMAPK and BraMAPK proteins.**

| Motif | Width | Sites | Group | e-value | Sequence |
| --- | --- | --- | --- | --- | --- |
| Motif 1 | 113 | 52 | ABCD | 1.0e-4696 | [NH][IV][SI]DA[KL]R[TI]LREIKLLR[HL][LM][RD]H[EP][ND][IV][VI][EA]IK[DH][IV][MI]LPPS[RK]REF[KR]D[VI]Y[VI]V[FY]ELM[DE][ST]DLHQ[VI]I[KR][SA]N[QD]DL[TS][PD][DE]H[CHY]Q[FY]FLYQ[LI]LRGLKY[IV]H[ST]AN[VI][LF]HRDLKP[KS]N[IL]L[AL]NA[ND]C[KD]LKICDFGLAR |
| Motif 2 | 113 | 23 | D | 1.9e-2242 | V[SA]FND[TA]P[TS][AT][IV]FWTDYVATRWYRAPELCGSF[FY]SKYTPAIDIWS[IV]GCIFAE[MV]L[TL]GKPLFPGK[NS]VVHQL[DE]L[MI]TD[LF]LGTP[SKP][PS]E[TA]IS[RG][VI]RN[ED]KAR[RK]YL[SG][SNE]MR[KR]K[PQ]P[VI]PF[ST][HQ]KFP[NKH]ADP |
| Motif 3 | 82 | 23 | D | 6.8e-1402 | LAL[RK]LL[QE]RLLAFDPKDRP[TS]AEEALADPYFKGL[AS][KN]V[ED]REPS[CT]QPI[ST]K[LM]EFEFER[RK][KR]LTK[DE]D[VI]RELIYREILEYHPQ[ML]L[KQE][ED]Y[LM][RN] |
| Motif 4 | 57 | 29 | ABC | 2.9e-1325 | [DQ]FMTEYVVTRWYRAPELLL[NC][CS][SD][END]Y[TG][ATS][AS]ID[VI]WSVGCIF[AMG]E[IL][ML][TG]R[KE]P[LI]FPG[KRT][DE][YCH][VL][HN]QL[RK] |
| Motif 5 | 29 | 52 | ABCD | 8.2e-1107 | [PV]IG[KR]G[AS]YG[VI]VC[SA]A[IV][DN][ST][EH]T[GN]E[KR]VAIKKI[NH][ND] |
| Motif 6 | 65 | 29 | ABC | 6.5e-1052 | [FG][LIS][RLD][NS][EDP][NK]A[KR]RY[IVL][RK][QS]LPY[YSF]P[RG]QS[FL][SA]A[LKR][FY]PN[VAM][NPS]PLAIDLL[EQ][KR]ML[VT]FDP[SR][KR]RI[ST]V[ED][ED]ALAHPY[LM][AS][PS]LHD |
| Motif 7 | 29 | 29 | BD | 2.1e-367 | [GQ][STF][SN]F[MLE]YPS[AG][VIT][DEG][HQRE][FL][KRI][KR][QE]FA[YH]LEE[NHV][SK][GF][KN][PG][GE]P |
| Motif 8 | 29 | 21 | AB | 4.4e-322 | V[LP][TS]H[GD]GR[YF][IV]QYN[VI][YF]GN[IL]FE[VL][TS][AR]KY[KV]PPI[RM] |
| Motif 9 | 29 | 17 | D | 5.70E-218 | M[QH][QP]DQ[QR]KK[NS]TKEM[DE]FFTEYG[DE]ANRY[QR]I[QL]E |
| Motif 10 | 29 | 15 | A | 4.90E-214 | EP[VE]CT[KIT]PF[NSD]FD[FL]E[EQ][HQ][PA]L[TDS]EEQ[IM]KELIYRE |
| Motif 11 | 21 | 23 | D | 4.40E-184 | [SV][PI][PA]L[EQ]R[KQ]H[AT]SLPR[ES][RT]V[PH][SA]SV[VN] |
| Motif 12 | 29 | 8 | C | 8.70E-154 | PNG[IV][RK][NQ][QE]GKHY[FY][ST]MWQTLFEIDTKY[VM]PIK |
| Motif 13 | 29 | 11 | D | 3.40E-134 | [AD][AN][GT][GP][GV][YK][SR][AS][RA][CNS]L[MV][KR]S[ADS]SI[SC][AG]S[KR]C[IV]GV[QS]S[KA][TV] |
| Motif 14 | 29 | 8 | C | 7.10E-105 | [IL][DS][LV]D[IEV]DE[ND][LM][EG][EA][ER]MIRE[ML]MW[NDE]EML[HY]YHP[EQ]A |
| Motif 15 | 15 | 20 | ABC | 2.20E-96 | LI[TI][EN][LIV][LVI]GS[PQ][DQ][ED][SE][DS]L[GE] |
| Motif 16 | 22 | 9 | D | 4.40E-68 | [KR][IMV][AGI]ID[AT][KN]LL[QH]A[QT][SA][QG][FIY]G[PV]A[GA]AAA |
| Motif 17 | 61 | 3 | D | 4.30E-62 | R[IV]P[IV]NVPQTIQGAAVARPGKVVGSVLRYNNCG[AE]ATGVEALEQQQRRMVRNP[AV][GA][AV][AS]QYPKR[TS] |
| Motif 18 | 35 | 9 | D | 7.30E-57 | [THS][AKT][TP][LI][GQ][PA][PQ][PQ]R[NV][PL][SP][SAG][GAR][KRP][PG][SGR][RAT][VF][VME][EGS][SP][SV][AV][PST][YF][ED]N[GN]RN[LS][KR][ED][AS] |
| Motif 19 | 29 | 10 | D | 3.60E-52 | [SYV][FV][HPR][ASP][QA][TVL][SV]S[PAS][SHN][CV][VYF]F[TK][PA][NP][TQ][MT][PMN][NQ][TEP][EAK][TKNR][GRS][LG][SG][EST][AKQ][KAS] |
| Motif 20 | 15 | 8 | D | 5.80E-39 | [VG][AS]AHR[KNS][VI]G[TA]V[GR]Y[GS][MA][SA] |
| Motif 21 | 15 | 6 | D | 1.80E-44 | FFTEYGEAS[QR]YQIQE |
| Motif 22 | 21 | 5 | D | 1.40E-38 | [MG]GGG[GS]NLVDG[VL][RL]RWLF[QF][RQ][PR][SR][SP] |
| Motif 23 | 29 | 3 | D | 3.00E-24 | EG[SP]SRLK[QP]N[PT]QYIPQKV[AGS][AG]AQDTA[MT]SRWY |
| Motif 24 | 21 | 5 | D | 7.40E-21 | [AE]E[ERS][SI][SN]D[VI]ERR[AIT][AST]A[AV]VASTL[ED]S |
| Motif 25 | 21 | 3 | D | 1.60E-19 | ARGGQPKLMNNTNTLNPETTQ |
| Motif 26 | 11 | 9 | A | 1.10E-14 | [LM]E[LF][IL]G[ST]P[ST]E[ED][ED] |
| Motif 27 | 18 | 3 | D | 2.40E-14 | TTME[SP]E[KR]QARQISQYNRY |
| Motif 28 | 21 | 3 | D | 5.00E-14 | PDVAINIDNNPFI[ML][AS]RTG[ML][HN]K |
| Motif 29 | 15 | 11 | BD | 1.20E-13 | [DS][GQ][EQ]S[SV][SA]E[LV]TD[GH][LCV][SI][KS][CLV] |
| Motif 30 | 29 | 4 | A | 5.00E-12 | [PT][NP][DN][ADN][AH][EV][PMT][VAG][EK]T[DN][AG][GR]ATTD[AL][QK][LP][HY]P[PS]P[EQ][NV][LIS][YAP][MV] |
| Motif 31 | 8 | 8 | AC | 1.20E-11 | [AS]NPPA[QH]VP |
