# Supplementary figures and images for: Genome-Wide Survey and Expression Profile Analysis of the Mitogen-Activated Protein Kinase (MAPK) Gene Family in Brassica rapa
Source: PLoS One. 2015 Jul 14;10(7):e0132051. doi: 10.1371/journal.pone.0132051 (PMC4501733; doi:10.1371/journal.pone.0132051)

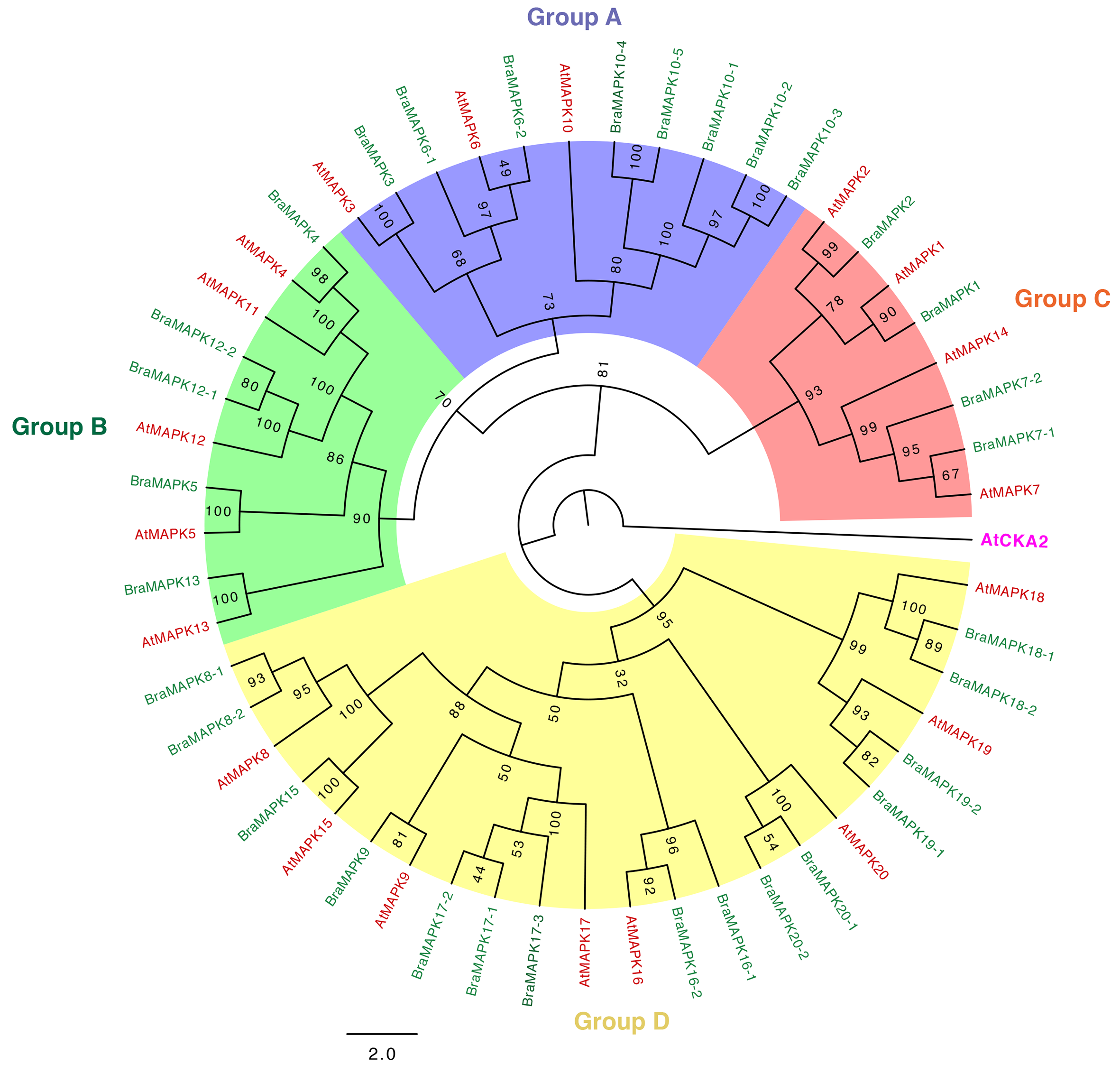

Supplement: S1 Fig — The phylogenetic tree was generated by the PhyML version 3.0.1 from alignment of amino acid sequences of 20 AtMAPKs, 32 BrMAPKs and AtCKA2, using the JTT model of amino acid substitution, an estimated gamma distribution parameter, and 100 bootstrap replicates. The tree was displayed with FigTree v1.4.0. At: Arabidopsis thaliana; Bra: B. rapa. (TIF) [file pone.0132051.s007.tif]

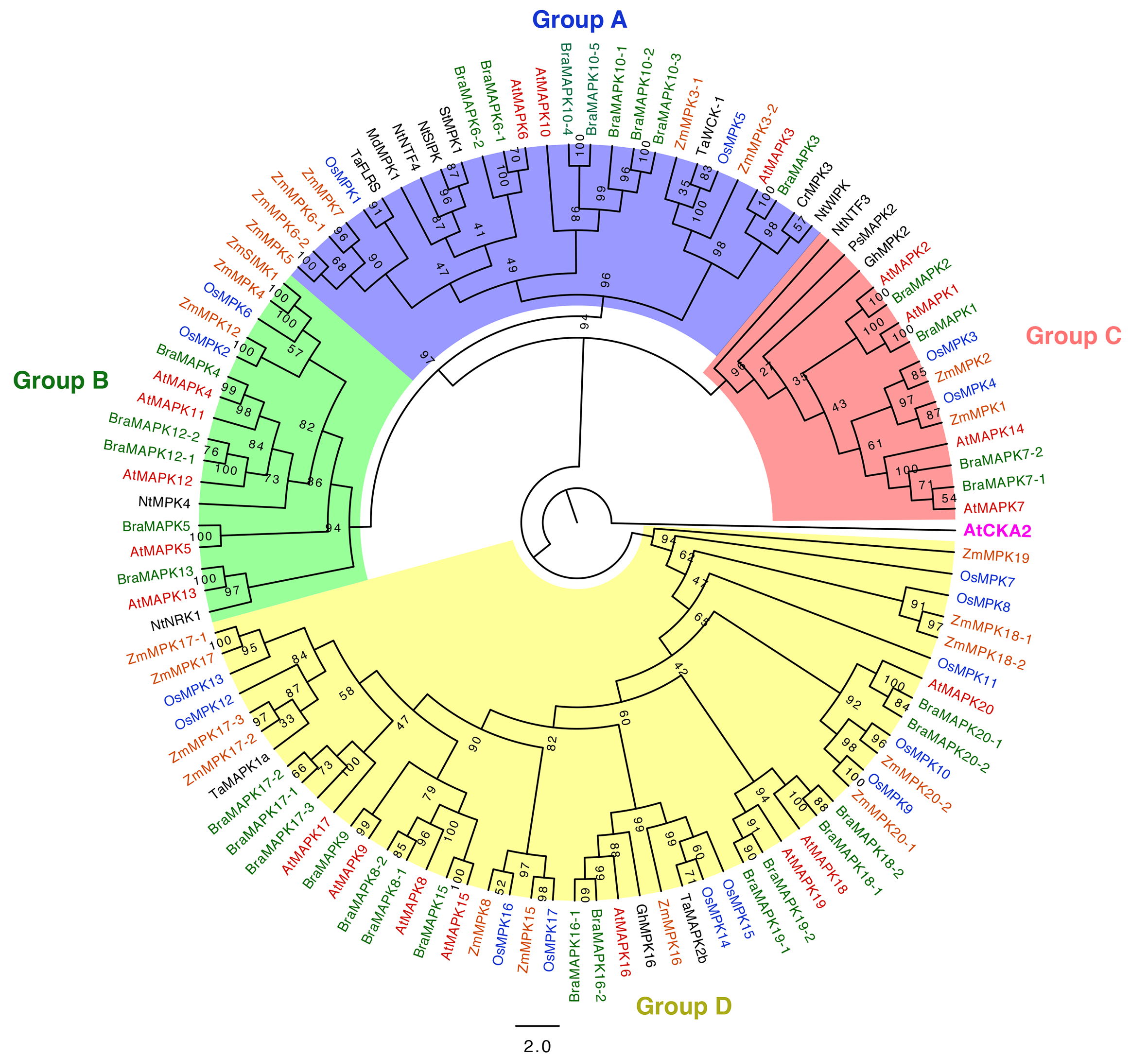

Supplement: S2 Fig — The phylogenetic tree derived by the ML method with bootstrap analysis (100 replicates) from alignment of amino acid sequences of MAPK proteins in Arabidopsis, B. rapa, rice, Zea mays, and other plants using PhyML version 3.0.1 program. The tree was displayed with FigTree v1.4.0. Only bootstrap values greater than 50% are denoted at the nodes. At: A. thaliana; Bra: B. rapa; Cr: C. roseus; Gh: G. hirsutum; Md: M. domestica; Nt: N. tabacum; Os: O. sativa; Ps: P. sativum; St: S. tuberosum; Ta: T. aestivum; Zm: Z. mays. (TIF) [file pone.0132051.s008.tif]

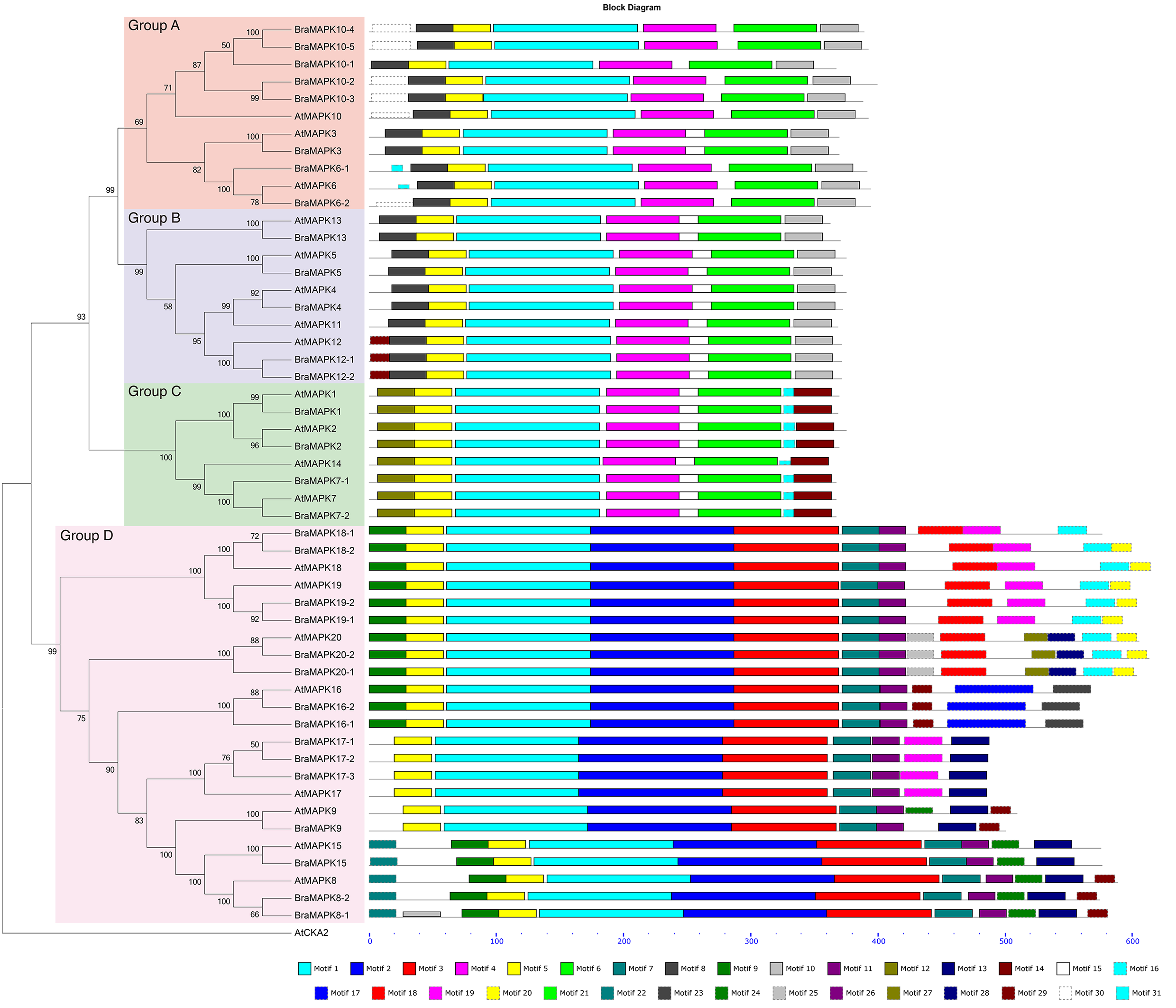

Supplement: S3 Fig — The phylogenetic tree (left panel) was generated from an amino acid sequence alignment of 20 AtMAPKs and 32 BrMAPKs using MEGA 6.0 with the ClustalW2 program and the NJ method (1000 bootstrap replicates), and displayed using FigTree v1.4.0. Only bootstrap values greater than 50% are denoted at the nodes. The 52 MAPK proteins in A. thaliana and B. rapa were clustered into four distinct groups (Groups A, B, C, and D). Distribution of conserved motif in AtMAPKs and BrMAPKs is shown in the right panel. A total of 31 motifs with an e-value of < 1e-10 were identified using the MEME program. Each motif is represented by a colored box. Box length corresponds to motif length. The specific lengths, groups, and e-values of each motif are listed in S3 Table. At: A. thaliana; Bra: B. rapa. (TIF) [file pone.0132051.s009.tif]

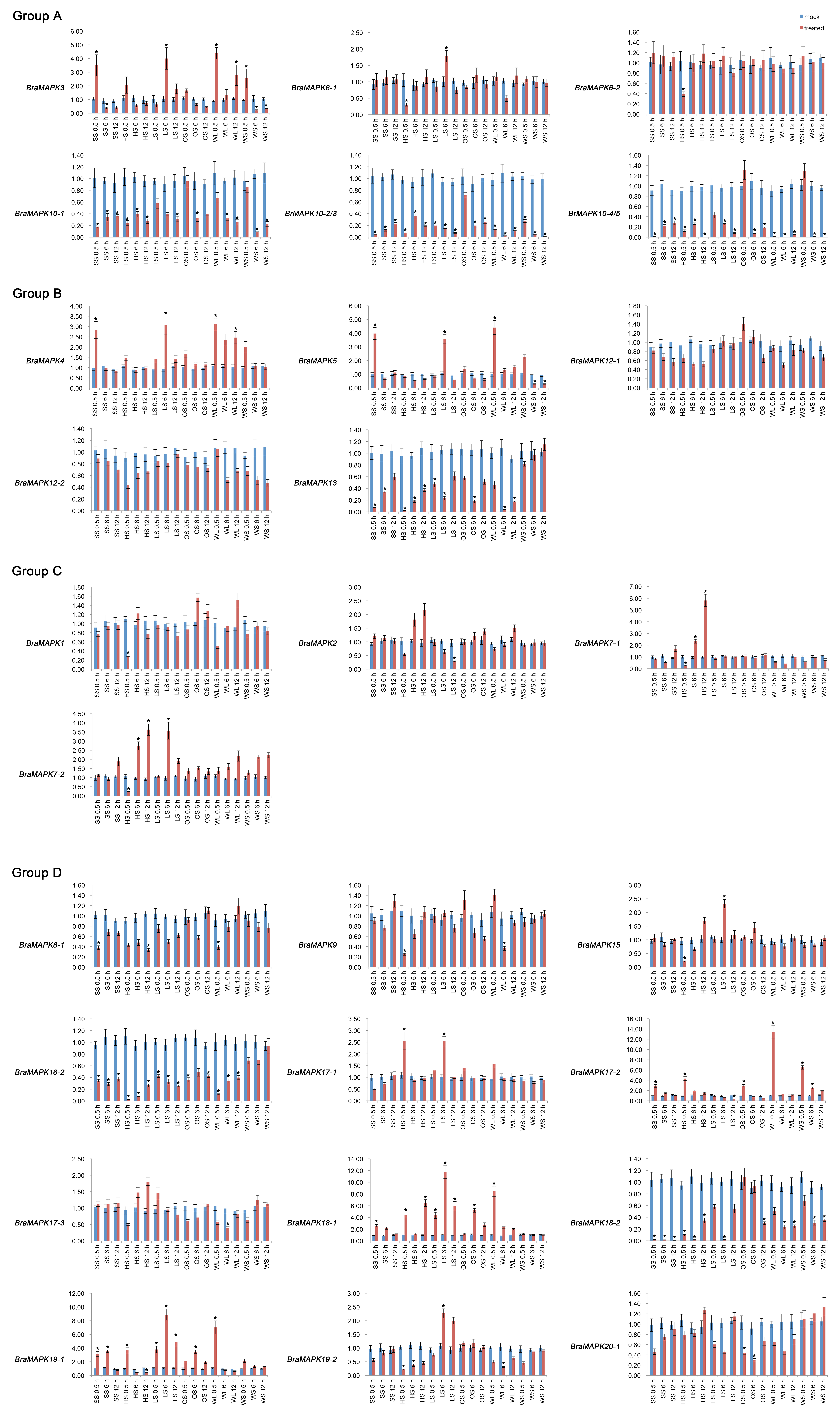

Supplement: S4 Fig — Transcription levels of BraMPAK genes were determined by qRT-PCR with gene-specific primers under salt (200 mM NaCl), heat (37°C), cold (4°C), osmotic (10% PEG-8000), waterlogging, and wound stresses. The expression levels were normalized against BraUBC21 and BraGAPDH genes using 2–ΔΔCT method [69]. Data are means ± SD (n = 3) and are representative of similar results from three independent experiments. Asterisk (*) on top of error bar indicate the significant difference (p-value < 0.05) compared with mock-treated controls. SS, HS, LS, OS, WL, and WS denote salt, heat, cold, osmotic, waterlogging, and wound stress treatments, respectively. (TIF) [file pone.0132051.s010.tif]

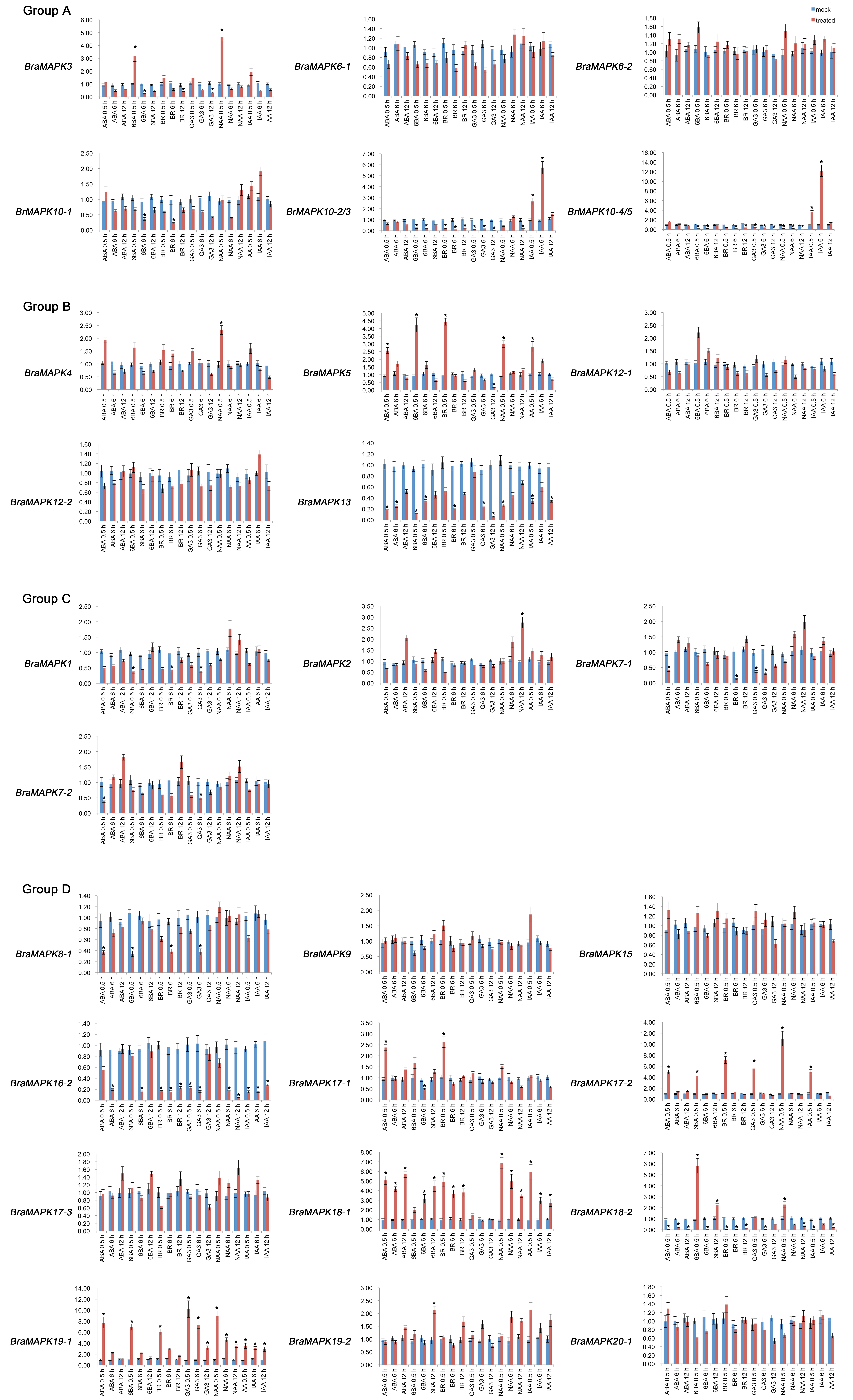

Supplement: S5 Fig — Expression levels of BraMAPK genes assayed by qRT-PCR under ABA (100 μM), 6-BA (10 μM), BR (2.5 μM), GA3 (25 μM), NAA (10 μM) and IAA (10 μM) hormone treatments. The expression levels were normalized against BraUBC21 and BraGAPDH genes using 2–ΔΔCT method [69]. Data are means ± SD (n = 3) and are representative of similar results from three independent experiments. Asterisk (*) on top of error bar indicate the significant difference (p-value < 0.05) compared with mock-treated controls. ABA: abscisic acid; 6-BA: 6-benzyladenine; BR: brassinolide; GA3: gibberellic acid; NAA: α-naphthaleneacetic acid; IAA: indole acetic acid. (TIF) [file pone.0132051.s011.tif]
